# Genetic diversity of currently circulating rubella viruses: a need to define more precise viral groups

**DOI:** 10.1099/jgv.0.000680

**Published:** 2017-04-01

**Authors:** P Rivailler, E Abernathy, J Icenogle

**Affiliations:** Division of Viral Diseases, Centers for Disease Control and Prevention, Atlanta, GA, USA

**Keywords:** rubella virus, circulating genotypes, evolution, geographic distance, genetic distance, Mantel test

## Abstract

Recent studies have shown that the currently circulating rubella viruses are mostly members of two genotypes, 1E and 2B. Also, genetically distinct viruses of genotype 1G have been found in East and West Africa. This study used a Mantel test to objectively include both genetic diversity and geographic location in the definition of lineages, and identified statistically justified lineages (*n*=13) and sub-lineages (*n*=9) of viruses within genotypes 1G, 1E and 2B. Genotype 2B viruses were widely distributed, while viruses of genotype 1E as well as 1G and 1J were much more geographically restricted. This analysis showed that more precise groupings for rubella viruses are possible, which should improve the ability to track rubella viruses worldwide. A year-by-year analysis revealed gaps in surveillance that need to be resolved in order to support the surveillance needed for enhanced control and elimination goals for rubella.

## Introduction

Rubella virus (RuV) is a positive-sense ssRNA virus in the genus *Rubivirus* within the family *Togaviridae*. The RuV genome is approximately 9762 nt in length and encodes five proteins, of which three are structural, the C or capsid and two envelope proteins, E1 and E2, and two are non-structural, P150 and P200.

Virological surveillance data on RuV are used to track progress towards goals for enhanced control and elimination, and possible eradication of rubella, to help with case classification, and to document transmission pathways. Although known RuVs comprise a single serotype, sufficient genetic variability exists to allow for virologic surveillance [1]. A systematic nomenclature for wild-type RuVs was established in 2006 [1, 2]. Updates to the nomenclature were reported in 2007 and 2013 [3, 4]. Currently, RuVs are divided into two clades (1 and 2), which differ by 8–10 % at the nucleotide level. The clades are sub-divided into genotypes, which are designated by letters, uppercase for accepted genotypes and lowercase for provisional genotypes. Clade 1 contains 10 genotypes (1a–1J), of which only 1a is provisional, and clade 2 contains 3 accepted genotypes (2A–2C). Initial phylogenetic studies on RuV were conducted using the sequences from all or most of the coding regions for the E1 protein, but a large majority of RuV sequences currently available are from a window of 739 nt (nucleotides 8731–9469) in the E1 coding region that was recommended by the World Health Organization (WHO) for routine molecular characterization [2]. In recent years, whole-genome sequencing has become more feasible [5–7], but the number of fully sequenced RuVs available in GenBank is still below 50. As new whole-genome sequencing techniques (so-called next-generation techniques) become more widely utilized, the availability of more whole genomes for RuVs will allow objective evaluation of other possible sequence windows.

Although 13 RuV genotypes are recognized, only 4 of the genotypes (1E, 1G, 1J and 2B) are now commonly detected [4, 8]. Genotype 2A was last detected in 1980, except for several 2A vaccine strain isolates, [9] and genotypes 1D and 1I were last detected in the 1990s. Genotypes 1B, 1C, 1F, 1H and 2C were still detected in the first decade of this century, but no viruses of these genotypes have been detected since 2010. Genotype 1a, which is composed mostly of RuVs from the 1960s, including most of the vaccine strains, has been detected sporadically; however, it is possible that some of these detections are due to laboratory contamination, since most of the commonly used laboratory strains are also genotype 1a viruses. Of the four currently active genotypes, 1E and 2B have a wide geographic distribution and are frequently detected, with genotype 2B being the most widely distributed. The remaining two genotypes, 1G and 1J, appear to be more restricted geographically and are less frequently detected. Together, the number of sequences from these four genotypes represents over 70 % of all the sequences available for analysis, and intra-genotype diversity as high as 2.6 % has been observed for viral sequences of genotype 1G . It is clear that if classification of currently circulating viruses into more than four genotypes was possible, it would enhance the utility of the molecular epidemiology of RuV. To this end, a dataset of 1109, 739 nt-long RuV sequences (E1 gene) has been created and analysed to determine if the data support further sub-division of RuV genotypes based on sequence diversity and/or geographical distribution. Results reported here provide support for division of viruses of genotypes 1E, 1G and 2B into several defined lineages.

## Results

### Genotype distribution in 2010–2014

Of the 1109 sequences in the full dataset, 635 represent viruses collected between 2010 and 2014. These 635 sequences represent viruses collected from 21 countries, with mainland China, Japan and Taiwan being the countries from which the most viruses were reported (Fig. S1, available in the online Supplementary Material). Genotype 2B is the most frequently represented (414 sequences) followed by 1E (198 sequences). Genotypes 1G and 1J are represented by only 17 and 6 sequences, respectively. Genotype 2B was reported in 20 countries on five continents. Genotype 1E was reported by nine countries, mainly from eastern Asia. Genotype 1J was rarely detected, with four countries reporting six sequences in total. Genotype 1G was reported by four countries, mainly from Africa. Epidemiologic data were available for 38 cases (Table S1).

Based on the available epidemiological data, RuVs of any genotype collected between 2010 and 2014 originated from at least 30 countries in Asia, Africa and Europe. A year-by-year analysis shows that at least 21 countries had clear molecular epidemiological evidence of circulation of rubella (Table 1), as RuV has been detected in at least 3 years of the last 5 or has been exported at least once. Among these countries, only China and Japan are consistently reporting virological data, with a mean of 40 and 30 virus sequences reported per year, respectively. In contrast, India, with a similar population size to China, reported only one viral sequence per year. Finally, there were no rubella genotypes from Africa reported in 2014, although rubella is endemic in many African countries based on incidence as determined by laboratory-confirmed cases [10] (Fig. S1). Concerning the genotype distribution, genotype 2B and 1E viruses were reported every year from 2010 to 2014 (Fig. S1). It is likely that detection of genotypes 1J and 1G is under-reported as they were predominantly found in countries with little or no rubella virologic surveillance.

**Table 1.** List of countries likely to be exporters of RuV

Country*	Number of years of collection	Known exportation
China	5	Yes
India	5	Yes
Japan	5	Yes
Hong Kong	5	
Vietnam	4	Yes
Thailand	4	
Indonesia	3	Yes
Philippines	3	Yes
Tunisia	3	
Uganda	3	
Democratic Republic of the Congo (DRC)	3	
Romania	2	Yes
Afghanistan	1	Yes
Algeria	1	Yes
Kenya	1	Yes
Nigeria	1	Yes
Pakistan	1	Yes
South Africa	1	Yes
Sudan	1	Yes
United Republic of Tanzania	1	Yes
Yemen	1	Yes

*Countries where rubella infection has been reported in at least 3 years between 2010 and 2014 or with at least one exported case between 2010 and 2014. Countries are listed based on decreasing number of years of collection.

### Phylogenetic analysis of the four circulating genotypes: 1J, 1G, 1E and 2B

Phylogenetic analysis of genotype 1J sequences showed that the six sequences of currently circulating viruses (identified with black circles in Fig. S2) cluster in one group that is part of a main lineage supported by a 69 % bootstrap value. The number of sequences available for genotype 1J is insufficient to further divide the genotype. Four of the six sequences were either detected in or known to be exported from the Philippines.

**Fig. 1.**
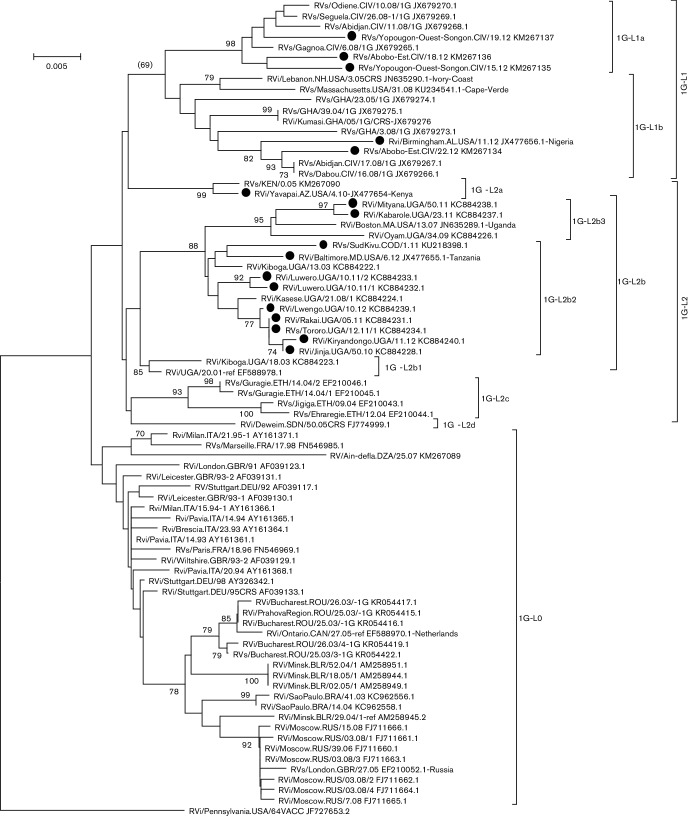
Phylogenetic tree of RuVs of 1G genotype. A neighbour joining tree was generated with mega 6 using the maximum composite likelihood nucleotide substitution model [20–22]. The phylogenetic inference was tested with the bootstrap method with 1000 replications. Bootstrap values greater than 70 % are shown. Sequences of viruses collected after 2010 are identified with a black circle. The tree was rooted with RVi/Pennsylvania.USA/64VACC_JF727653.2.

The number of sequences of genotype 1G is limited (77 sequences, 17 since 2010), but the genetic diversity between these sequences has a maximum of 5.4 % nucleotide divergence and an average divergence of 2.9 %. Fig. 1 illustrates the phylogenetic analysis, and Fig. S3 describes the analysis process for 77 genotype 1G sequences. Genetic distance matrices and geographic distance matrices for these 77 sequences were compared using a Mantel test (see Methods). The Mantel test showed a correlation between genetic and geographic distances (*P*=1×10^−4^). The topology of the tree identified three groups, 1G-L0, 1G-L1 and 1G-L2 (Table 2, Fig. 1). 1G-L0 is represented by sequences of viruses mainly collected in Europe between 1991 and 2008 and not currently known to be circulating. 1G-L1 is composed of sequences from viruses collected in or exported from West Africa, mostly Republic of Côte d’Ivoire but also Ghana, Nigeria and Cape Verde. This group was further divided into two sub-groups (1G-L1a and 1G-L1b) because of a strong bootstrap value (1G-L1a is supported by 98 % bootstrap) and co-circulation (RVs/Abobo-Est.CIV/18.12 of 1G-L1a and RVs/Abobo-Est.CIV/22.12 of 1G-L1b). 1G-L2 viruses, found in East Africa, are characterized with significant nucleotide diversity within the 739 nt sequencing window, with a mean intra-lineage distance greater than 2 %. Also, the Mantel test showed that the 1G-L2 sequences are geographically clustered (*P*=1×10^−4^). Based on the topology of the phylogenetic tree, we defined four groups: 1G-L2a found in Kenya, 1G-L2b found in Uganda, 1G-L2c found in Ethiopia and 1G-L2d found in Sudan. 1G-L2a and 1G-L2c are supported by bootstrap values of 99 and 93 %, respectively. A 1G-L2a virus was detected in Kenya in 2005. However, another 1G-L2a virus collected in the USA in 2010 (RVi/Yavapai.AZ.USA/4.10-JX477654) was also imported from Kenya, suggesting that rubella is endemic in Kenya. Lineage 1G-L2b is represented by viruses from Uganda. This sub-lineage showed some genetic diversity (with mean intra-group distances close to 1.5 %) and was further divided into three groups in a recent publication, where they were called ‘lineages 1, 2 and 3’ [11]. To be consistent with the proposed designations, these lineages were identified as 1G-L2b1, 1G-L2b2 and 1G-L2b3. The 1G-L2b1 and 1G-L2b3 lineages are supported by bootstrap values greater than 85 %. Lineage 1G-L2b1 is likely to be extinct, whereas viruses of 1G-L2b2 and 1G-L2b3 are co-circulating. RVi/Mityana.UGA/50.11-KC884238 (1G-L2b2) was collected in the Luwero district, where RVi/Luwero.UGA/10.11/2-KC884233 (1G-L2b3) was also reported [11]. Lineage 1G-L2c viruses, detected in Ethiopia, have not been reported since 2004. No rubella case has been linked to Ethiopia since 2004, so it is not possible to know if the 1G-L2c lineage is extinct or not detected due to a lack of adequate surveillance. Lineage 1G-L2d is an orphan with only one virus, collected in Sudan, RVi/Deweim.SDN/50.05CRS (FJ774999). The last time a RuV was reported from Sudan was in 2006 (RVs/Gadarif.SDN/22.06CRS-FJ774998). However, an imported congenital rubella syndrome (CRS) case of genotype 1E from Sudan identified in 2012 in the USA (RVs/Chicago.IL.USA/38.12 [1E] CRS-KC866357) as well as surveillance data www.emro.who.int/vpi/publications/measles-monthly-bulletin.html) show that rubella is endemic in Sudan. We expect that the designation of RuV lineages will be ongoing. A full description of the lineages circulating in Sudan will require many more viruses.

**Table 2.** Summary of the analysis

Genotype		Lineages						Description†		Representative virus‡		‘Within-group’ mean genetic distance	‘Between group’ smallest mean genetic distance	Reference
Name	No.	Primary	No.*	Secondary	No.	Tertiary	No.	Time period	Geographic source	WHO name	GB-ID			
1G (g)§	77 (17)	L0	36					1991–2008	10 countries (Europe, North Africa, South America)	RVi/Ontario.CAN/27.05-REF_Netherlands	EF588970	0.0149	0.0219	
		L1*	17 (5)	L1a (b)*,§	7 (3)			2008–2012	Ivory Coast	RVs/Gagnoa.CIV/6.08	JX679265	0.0125	0.0289	
				L1b*	10 (2)			2004–2012	Ghana, Ivory Coast, Nigeria, Cape Verde	RVs/GHA/39.04	JX679275	0.0205||	0.0250	
		L2 (g)§	24 (12)	L2a (b)§	2 (1)			2005–2010	Kenya	RV/KEN/0.05	KM267090	0.0054	0.0198	
				L2b	17 (11)	L2b1 (b)§	2	2001–2003	Uganda	RVi/UGA/20.01-REF	EF588978	0.0068	0.0178	[11]
						L2b2*	11 (9)	2003–2012	Uganda, Tanzania, DRC	RVi/Kiboga.UGA/13.03	KC884222	0.0093	0.0178	
						L2b3 (b)*,§	4 (2)	2007–2011	Uganda	Rvi/Boston.MA.USA/13.07_Uganda	JN635289	0.0136	0.0215	
				L2c (b)§	4			2004	Ethiopia	RVs/Jigiga.ETH/09.04	EF210043	0.0099	0.0198	
				L2d	1			2005	Sudan	RVi/Deweim.SDN/50.05CRS	FJ774999	n/c	0.0198	
1E (g)§	460 (198)	L0	96					1995–2008	17 countries (Europe, Americas, Africa, Asia)	RVs/Caen.FRA/23.95	FN546967	0.0112	0.0189	
		L1*	306 (161)					2001–2014	China, Taiwan, Japan, Russia, Hong Kong, Vietnam	RVI/DEZHOU.CHN/02-REF	KF201674	0.0127	0.0189	[14]
		L2 (b)*,§	47 (29)					2001–2014	Taiwan, Japan, Malaysia, Kazakhstan, Indonesia, China, Hong Kong	RVi/MYS/01-REF	AY968221	0.0152	0.0252	
		L3 (b)*,§	7 (5)					2008–2011	Tunisia	RVi/Ariana.TUN/08.08	KF018682	0.0114	0.0288	
		L4 (b)*,§	4 (3)					2005–2013	Yemen, Sudan, Uganda	RVi/Deweim.SDN/24.05CRS	FJ775000	0.01738||	0.0240	
2B (g)§	542 (414)	L0*	72 (3)					1992–2010	14 countries (Europe, Americas, Africa, Asia)	RVi/Milan.ITA/42.94	AY161370	0.0127	0.01446¶	[12]
		L1*	354 (347)					2008–2014	14 countries (Asia, Europe)	RVs/HongKong.CHN/49.08	FJ656218	0.0073	0.01446¶	
		L2 (g)*,§	94 (61)	L2a*	25 (3)			2000–2013	South Africa, India, Pakistan, Kazakhstan, Taiwan	RVi/Seattle.WA.USA/16.00-REF_India	JN635293	0.0113	0.0265	
				L2b (b)*,§	11 (5)			2005–2011	India, Thailand, Japan, Taiwan, USA	RVi/Tamilnadu.IND/20.05CRS	KC618672	0.0082	0.0279	
				L2c (b)*,§	58 (53)			2005–2014	15 countries (Europe, Africa, Asia)	RVs/Kerala.IND/42.05CRS	KC618675	0.0119	0.0305	
		L3 (b)*,§	14 (3)					2006–2010	Taiwan, Vietnam, China	RVi/Chendu.Sichuan.CHN/18.06/1	FJ875057	0.0092	0.0409	
		L4 (b)§	8					1995–2008	South Korea, China	RVi/Anqing.Anhui.CHN/00-ref	AY968218	0.0197||	0.0344	

No. represents number of analysed sequences (only viruses with true geographic source were included in the analysis); DRC, Democratic Republic of the Congo.

*Lineages containing sequences reported since 2010. Number of sequences from the 2010–2014 time period are indicated in parentheses.

†‘Time period’ and ‘geographic source’ are based on the phylogenetic trees.

‡The representative virus is either a WHO reference virus or the ‘oldest’ virus collected.

§Criteria for grouping: (b): bootstrap>70 %; (g): Mantel test, *P*=1×10^4^.

||‘Within-group’ mean genetic distance >1.5 %. There are not enough data to break the groups at the time of the analysis.

¶Smallest ‘between-group’ distance <1.5 %. Although the genetic distance is small between these two groups, these groups are kept to differentiate between currently circulating viruses and those collected at an earlier time.

Table 2 summarizes the various lineages and their characteristics. Genotype 1G is geographically clustered [noted with (g)] and is divided into three primary lineages: 1G-L0, 1G-L1 and 1G-L2. Lineage 1G-L1 is supported by a bootstrap value greater than 70 %, and lineage 1G-L2 is geographically clustered. Both of these lineages comprise viruses collected after 2010. Lineage 1G-L1 is further divided into two secondary lineages; both are supported by a bootstrap value greater than 70 %. Lineage 1G-L2 is divided into four secondary lineages, two of which are supported by a bootstrap value greater than 70 %. Finally, lineage 1G-L2b is further divided into three tertiary lineages, two of them being supported by a bootstrap value greater than 70 %. Mean pairwise genetic distances within and between groups were computed to validate the ultimate groupings. All groups are validated, except 1G-L1b, which shows a within-group mean pairwise distance of 2 %. However, there are not enough data to further split 1G-L1b.

Genotype 1E viruses have been collected since 1995 in 24 countries representing all continents except Antarctica. Four hundred and sixty sequences have been analysed, and 42 % of these sequences are from viruses reported since 2010 and collected in 10 countries located mainly in Asia. This demonstrates that 1E is still circulating but in a limited geographical area. Genotype 1E sequences are highly divergent; the most divergent sequences show 5 % nucleotide diversity. Genotype 1E sequences have an average pairwise nucleotide divergence of 2 %. A Mantel test showed that the 460 1E sequences are geographically clustered (*P*=1×10^−4^). Based on the topology of the phylogenetic tree, five clusters were established, called 1E-L0, 1E-L1 mainly in China, 1E-L2 mainly in East Asia, 1E-L3 mainly in Tunisia and 1E-L4 in East Africa and Western Asia (Fig. S4). Lineage 1E-L0 is paraphyletic and likely extinct. Lineage 1E-L1 contains recent viruses, predominantly from China: 52% (161 of 307 sequences) of lineage 1E-L1 sequences are sequences of viruses collected since 2010, with 88 % of 1E-L1 lineage viruses collected in mainland China. An additional 5 % are from viruses identified as exportations from mainland China [12]. The remaining 20 sequences are from viruses collected in Taiwan, Russia and Japan. Epidemiologic data are missing in these cases, but it is reasonable to presume that these viruses originated from mainland China as well. A Mantel test on lineage 1E-L1 showed no geographic clustering (*P*=0.2765), confirming a unique geographical source, China. Lineages 1E-L2, 1E-L3 and 1E-L4 are supported by bootstrap values greater than 78 %. The GenBank dataset ending in 2014 used here showed that lineage 1E-L2 is a heterogeneous group meeting the proposed criterion for sub-division (within lineage distance of 1.52 %). Twelve of the 14 *sequences* since 2010 are clustered in one sub-group supported by a bootstrap value of 72 %. Although we decided that it was premature to define a sub-lineage for 1E-L2, as sequences of more recent (and more divergent) viruses are determined, justification for further sub-division of the 1E-L2 will likely increase. Lineage 1E-L3 seems to be only circulating in Tunisia. Lineage 1E-L4 is characterized by four sequences, three of them from viruses collected since 2010. Two of these viruses were detected in the USA, but epidemiologic data showed that they are coming from Yemen and Sudan. A full description of the lineages circulating in Yemen and Sudan would require many more viruses from these countries/regions.

Genotype 2B is more divergent than the 1E sequences, with 6.7 % nucleotide diversity between the most divergent sequences. Phylogenetic analysis identified three sequences (Rvi/Iran/00_DQ975202.1, Rvi/Milan.ITA/22.93_AY161362.1 and RVi/TelAviv.ISR/68_AY968219.1) that are highly divergent from the other 2B sequences, with 5 % divergence compared to all the other sequences. These three sequences were considered outliers in this analysis, and they were not included in the analysis of the 2B genotype. Genotype 2B sequences were reported since 1968 in 25 widely distributed countries. Almost 75 % of the 542 sequences analysed (414) are from viruses collected since 2010 in 19 countries. A Mantel test on the 542 sequences identified a significant geographic clustering (*P*=2×10^−4^). Five groups were established based on the topology of the phylogenetic tree (Fig. S5). They were called 2B-L0, 2B-L1, 2B-L2, 2B-L3 and 2B-L4. The distance between groups was greater than 2.5 %, except for the distance between 2B-L0 and 2B-L1 (1.45 %). Although the distance is less than the 1.5 % cutoff, the distinction between lineages 2B-L0 and 2B-L1 is useful in that it allows focusing on the sequences of viruses collected since 2010. Lineage 2B-L0 is paraphyletic and likely extinct. Only six sequences of 2B-L1 were from viruses collected before 2010, the oldest sequence being from 2008. Lineage 2B-L1 is not geographically clustered (*P*=0.0164). These sequences are very homogeneous with a within-group distance of 0.7 %. In contrast, lineage 2B-L2 is genetically diverse with a within-group distance of 1.8 %. A Mantel test demonstrates some geographic clustering (*P*=1×10^−4^) within the 94 2B-L2 sequences. Three groups were established based on tree topology, called 2B-L2a, 2B-L2b and 2B-L2c. 2B-L2b and 2B-L2c are supported with bootstrap values greater than 80 %. All three 2B-L2 sub-lineages are likely to share a common ancestor originating from India, but epidemiologic and sequence data are insufficient to formerly assess the genetic origin of these lineages. Lineages 2B-L3 and L4 are mainly from eastern Asia.

## Discussion

To gain further insight into the genetic diversity of the currently circulating RuVs, we created a robust sequence dataset and analysed per cent identity, tree topology and geographical distribution. We found that for three of the four currently circulating genotypes, 1E, 1G and 2B, sufficient data exist to propose sub-dividing these genotypes into lineages, some of which also have evidence for geographic localization. The large number of sequences of genotypes 1E (460) and 2B (545), in particular, makes the genotype level designation alone not useful for molecular epidemiological purposes. For example, identifying a virus to be of genotype 2B is not very informative, as 2B viruses have a wide geographic distribution. However, assessing that a virus belongs to the 2B-L2 or L3 lineage defined here conveys more information in terms of location and time. Countries that are utilizing molecular surveillance to identify and document endemic RuVs and to understand the transmission of viruses during outbreaks would benefit from a standardized nomenclature of sub-divided genotypes that would enable clearer descriptions of the genetic diversity of their RuV sequences. In fact, this has been described in the literature, but in a non-standardized fashion. For genotype 2B, Tran *et al.* reported that in a large outbreak that occurred in Vietnam in 2009–2010, two distinct lineages of 2B were found [13]. Cheng *et al.* reported that viruses of genotype 2B collected in Taiwan between 2005 and 2011 could be divided into three lineages [14]. For genotype 1E, Zhu *et al.* described two clusters within genotype 1E viruses from China, designated as clusters 1 and 2 [15]. Cheng *et al.* examined genotype 1E viruses from Taiwan over a 7-year period and identified three lineages within 1E designated lineages 1, 2 and 3 [14]. One or more of these lineages may be the same clusters identified by Zhu *et al.* [15], but without standardized naming, this is difficult to determine. Another example is the three lineages of viruses of genotype 2B suggested by Namuwulya *et al.* [11]. The three lineages were found within one branch of a 1G-only phylogenetic tree and would, thus, be classified as sub-lineages using the naming strategy proposed here.

To help with future data analysis and definition of meaningful lineages, we described here a method for defining lineages. Although more than 2000 sequences of RuVs are available in GenBank, curation of the sequences was necessary to construct a robust dataset, mainly by elimination of duplicate sequences and multiple sequences derived from single outbreaks. Therefore, we chose to analyse two types of sequences. All unique sequences (unless they were found to contain errors such as deletions in coding regions or stop codons) and identical sequences from viruses were included only if they were collected at a different location or at least 2 weeks apart. Only sequences of currently circulating genotypes were analysed here, since these sequences are useful for molecular epidemiology. In this context, we did not analyse sequences of viruses belonging to genotypes that have not been reported for at least 10 years. Phylogenetic analysis was performed, and the resulting tree topology was examined to identify potential lineages. Since virologic surveillance for RuV is very incomplete, some expert opinion had to be used to organize sequences for analysis. For example, in a lineage composed of 10 sequences of viruses collected in West Africa and one sequence collected in the USA, where rubella has been eliminated, if there were no epidemiologic data directly linking the virus to West Africa, this sequence was deleted from the geographic analysis.

In order to better classify sequences belonging to the major genotypes, we suggest using three criteria: genetic diversity, geographic distribution and time. In general, the threshold for the level of genetic diversity within and between lineages is not standardized. We propose that a genetic distance of 1.5 % should be the cutoff for RuV sequences. Sequences within a lineage would differ by 1.5 % or less, while the distance between lineages would be greater than 1.5 %. The geographic distribution should be assessed in conjunction with genetic diversity. For example, a group of sequences could be genetically related by <1.5 %, but they could be distinguished by the location (i.e. where the group of viruses is circulating). The Mantel test compares genetic distance and geographic distance matrices. If these two matrices are correlated (i.e. if distant sequences are from viruses collected at distant locations and if close sequences are from viruses collected at close locations), then the *P* value from the Mantel test is very low (1×10^−4^). The Mantel test is often used in ecology to test the correlation between two types of distances [16]. We chose the Mantel test as it provides a statistic to measure objectively the likelihood of geographic clustering within a particular lineage. Finally, it is useful to identify genetically diverse viruses that are co-circulating.

Sequence data are much more useful if linked to meaningful metadata, and it is important that the metadata are as precise as possible. For example, knowing only the name of the country of detection is often not informative enough. Thus, it would be better to collect data at the smallest acceptable administrative level to estimate accurate latitude and longitude coordinates, which are required input data for the Mantel test computation. In addition, some lineages exist at a regional level (such as 2B-L1, found mainly in East Asia). Considering latitude/longitude coordinates would help to analyse such lineages, as national borders are not very meaningful in the context of regional distribution. The metadata most useful for sequence analysis are often not the same as the metadata most useful for public health purposes. For example, sequences of viruses imported into a country may be useful to that country, but these sequences are best linked to the geographic origin of the virus when defining viral lineages.

The present analysis was somewhat limited by major gaps in surveillance for RuV. Rubella was declared to be eliminated in the Americas in 2015 (www.paho.org); thus, surveillance in the Americas should be maintained, as these countries serve as sentinels for the entire world. For example, an importation into the USA from Kenya provided evidence for endemic circulation of 1G-L2a in Kenya. Many countries where rubella is known to be endemic, based on incidence rates [10], are not reporting molecular data for RuV [17]. As more countries adopt elimination goals and approach elimination, surveillance for rubella, including molecular characterization, will hopefully improve. Regional and national commissions responsible for verification of rubella elimination require virologic surveillance data showing lack of an endemic genotype, which is consistent with elimination.

The aim of this study was to better characterize sequences of RuVs belonging to the four currently circulating genotypes. Our analysis identified several interesting points. Genotype 1G, even though not as geographically widespread as genotypes 1E and 2B, is highly diverse, with distinct lineages localized in different regions of Africa. Clearly defining two lineages (here called 1G-L1 and 1G-L2) should be important in tracking viruses. Genotypes 1E and 2B are currently the most frequently detected. The proposed designations identify the main lineages (1E-L1 and 2B-L1, comprising 65 % of all 1E or 2B sequences) but also secondary lineages that could be useful to tracking viruses such as the 1E-L3 localized in Tunisia. Finally, this analysis identified three sequences, RVi/Iran/00_DQ975202.1, RVi/Milan.ITA/22.93_AY161362.1 and RVi/TelAviv.ISR/68_AY968219.1, that were previously genotyped as 2B viruses but display sufficient diversity from all other 2B viruses to justify removing them from the 2B genotype. As these three sequences are from viruses collected from 16 to 48 years ago and no similar viruses have been found since, it is not necessary to create a new genotype for these outliers. Newly sequenced viruses could be assigned to a genotype and lineage using standard methods such as phylogenetic analysis of the new sequence aligned with a set of reference viruses encompassing all the lineages. The addition of a new level(s) of RuV sequence designations could greatly improve the utility of molecular epidemiology in support of rubella control and elimination goals.

When many more RuV sequences from most geographic regions are available (1000s of sequences for each genotype), the work described here is expected to be a part of a systematic phylogeographic analysis of RuVs, as has been done for other viruses [18]. If any currently rare RuV genotype experiences a resurgence, then sufficient viruses for that genotype may be available to be a part of this systematic phylogeographic analysis.

## Methods

### Dataset

Thirty-eight sequences from five laboratories in the WHO Global Measles and Rubella Laboratory Network and not previously in GenBank were shared with Centers for Disease Control and Prevention (CDC) and used with permission in this analysis. GenBank accession numbers for all sequences are included in figures.

Sequence data were downloaded from GenBank [www.ncbi.nlm.nih.gov/nuccore/?term=txid11041(Organism : exp)] on 4 January 2016. Only sequences of viruses collected before 1 January 2015 were considered for the analysis. Furthermore, only sequences of viruses belonging to the currently circulating genotypes, i.e. 1E, 1G, 1J and 2B, were analysed. In total, 1369 sequences were aligned in BioEdit (www.mbio.ncsu.edu/BioEdit/bioedit.html) using the Muscle alignment tool [19]. The alignment window was set to the 739 nt window within the E1 ORF used to genotype RuVs (2005). Duplicated sequences were removed only if they were from viruses collected at the same location and at the same time (epi week ±2). The exporting country was determined based on epidemiologic data, including, but not limited to, travel history and country of birth. Sequences of viruses for which importation was suspected, but with no epidemiologic information, were removed from the analysis (e.g. a new genotype appearing in a country with established RuV surveillance that had not detected that genotype previously but without further information). The final dataset contained 1109 sequences. The annotated dataset (with phylogenetic groupings) is available upon request.

### Maps

Maps were generated in QGIS version 2.14.0. Shape files were downloaded from www.gadm.org/. Latitude and longitude were determined with www.findlatitudeandlongitude.com/. In cases where different administration levels had identical names (for example, Chinese province and city), the lowest administrative level (i.e. city) was used to determine the latitude and longitude.

### Phylogeny and nomenclature

Phylogenetic trees were generated with mega 6 using the neighbour joining method and the maximum composite likelihood nucleotide substitution model [20–22]. Trees with a similar topology to those presented were also obtained with the maximum likelihood method. The phylogenetic inference was tested with the bootstrap method with 1000 replications. Bootstrap values greater than 70 % are shown. Trees were rooted with RVi/Pennsylvania.USA/64VACC_JF727653 (the RA27/3 vaccine strain). Pairwise distances as well as mean distances within and between groups were computed in mega 6. The Mantel test was performed with the package ade4 in R (http://cran.r-project.org/web/packages/ade4/ade4.pdf) with 10 000 permutations. This test measures the correlation between genetic distance and geographic distance matrices. The lower the *P* value, the better the correlation between matrices and the higher the significance of geographic clustering. A significant *P* value is expected to be in the order of 1×10^−4^. Lineages were identified based on tree topology depending on genetic distances, geographic distances and co-circulation. Criteria for the genetic distance within a lineage were less than 1.5 %, and the distance between lineages was more than 1.5 %. These criteria are in agreement with previous nomenclature studies such as for influenza H5N1 [23]. The correlation between genetic distances and geographic distances was assessed with the Mantel test; a matrix of pairwise genetic distances was compared with a matrix of pairwise latitude/longitude coordinates based on the WHO name of the virus. Co-circulation was considered to have occurred when viruses differing by approximately 1.5 % at the nucleotide level were collected at the same time and at the same location (based on the WHO name of the virus). Mean pairwise genetic distances within and between groups were computed to validate the ultimate groupings.

By applying these criteria to three of the now commonly detected genotypes (1E, 1G and 2B), three levels of virus sequence groupings within some genotypes could be established (see Results). A systematic nomenclature was adopted to describe the grouping established here and to accommodate future groups that are likely to result from analysis of a more complete set of RuV sequences. A lineage, the first grouping below genotype, is designated by the genotype followed by a dash, L (for lineage) and a number (for example, 1G-L1). Lineages designated as 0 represent lineages that are not currently circulating and are likely extinct. If necessary, sub-lineages are identified by the lineage of which they are part followed by a letter and, if necessary, a number to designate a sub-sub-lineage. For example, 1G-L2b1 would represent the sub-sub-lineage 1, part of sub-lineage b, part of lineage 2 of genotype 1G.

## Supplementary Data

1377 Supplementary File 1
