# Supplementary material for: Genetic diversity of currently circulating rubella viruses: a need to define more precise viral groups
Source: J Gen Virol. 2017 Apr 1;98(3):396–404. doi: 10.1099/jgv.0.000680 (PMC5797949; doi:10.1099/jgv.0.000680)
Supplement: Supplementary File 1 [file jgv-98-396-s001.pdf]

Legend of the Supplementary Figures

Supplementary Figure 1:

Geographic distribution of rubella virus genotypes based on collection countries and likely-source countries between 2010 and 2014. The top panels show the distribution for the period 2010-2014 period. The panels below show the distribution year per year. The number of sequences analyzed by country as well as the genotype is color coded according to the legends shown at the bottom of the figure. Pie charts show the genotype distribution for each country.

Supplementary Figure 2

Phylogenic tree of rubella viruses of 1J genotype: a neighbor joining tree was generated with Mega 6 using the maximum composite likelihood nucleotide substitution model (Kumar *et al.*, 2004; Saitou & Nei, 1987; Tamura *et al.*, 2011). The phylogenetic inference was tested with the bootstrap method with 1000 replications. Bootstraps values greater than 70% are shown. Sequences of viruses collected after 2010 are identified with a black circle. The tree was rooted with RVi/Pennsylvania.USA/64VACC_JF727653.2.

Supplementary Figure 3:

Analysis workflow of genotype 1G. The analysis process leading up to the designation of primary, secondary and tertiary lineages within the 1G genotype is described. Reasons for splitting a genetic group are boxed and resulting groups are circled. All the relevant information is summarized in Table 2. * The tertiary lineages 1G-L2b1, 2 and 3 collected Uganda were previously described by Namuwulya et al. (Namuwulya *et al.*, 2014)

Supplementary Figure 4

Phylogenic tree of rubella viruses of 1E genotype, as indicated in Supplementary Figure 2.

Supplementary Figure 5

Phylogenic tree of rubella viruses of 2B genotype: as indicated in Supplementary Figure 2.

Supplementary Table 1: List of known imported cases

Collection Country	Source Country	Year	Genotype	CRS *
Taiwan	China	2010	1E	
Taiwan	China	2010	1E	
Taiwan	China	2010	1E	
Taiwan	China	2010	1E	
Taiwan	China	2010	1E	
United States	India	2010	2B	
United States	Kenya	2010	1G	
Hong Kong	Philippines	2010	1J	
Russia	China	2011	1E	
Taiwan	China	2011	1E	
Taiwan	China	2011	1E	
Taiwan	China	2011	1E	
Taiwan	China	2011	1E	
Taiwan	China	2011	1E	
Chile	India	2011	2B	
United States	India	2011	2B	
United States	Indonesia	2011	1E	
Hong Kong	Indonesia	2011	2B	
Japan	Philippines	2011	1J	
Canada	Philippines	2011	2B	
United States	South Africa	2011	2B	
Taiwan	Vietnam	2011	1E	
United States	India	2012	2B	
United States	Indonesia	2012	1E	
United States	Nigeria	2012	1G	yes
United Kingdom	Romania	2012	2B	
United States	Sudan	2012	1E	yes
United States	Tanzania, United Republic of	2012	1G	yes
United States	Algeria	2013	2B	
Canada	Japan	2013	2B	
United States	Japan	2013	2B	
United States	Pakistan	2013	2B	
United States	Yemen	2013	1E	yes
United States	Afghanistan	2014	2B	
United States	China	2014	2B	
United States	India	2014	2B	
United States	Indonesia	2014	1E	
Canada	Philippines	2014	1J	
*: sequence obtained from a case of congenital rubella syndrome (CRS)				

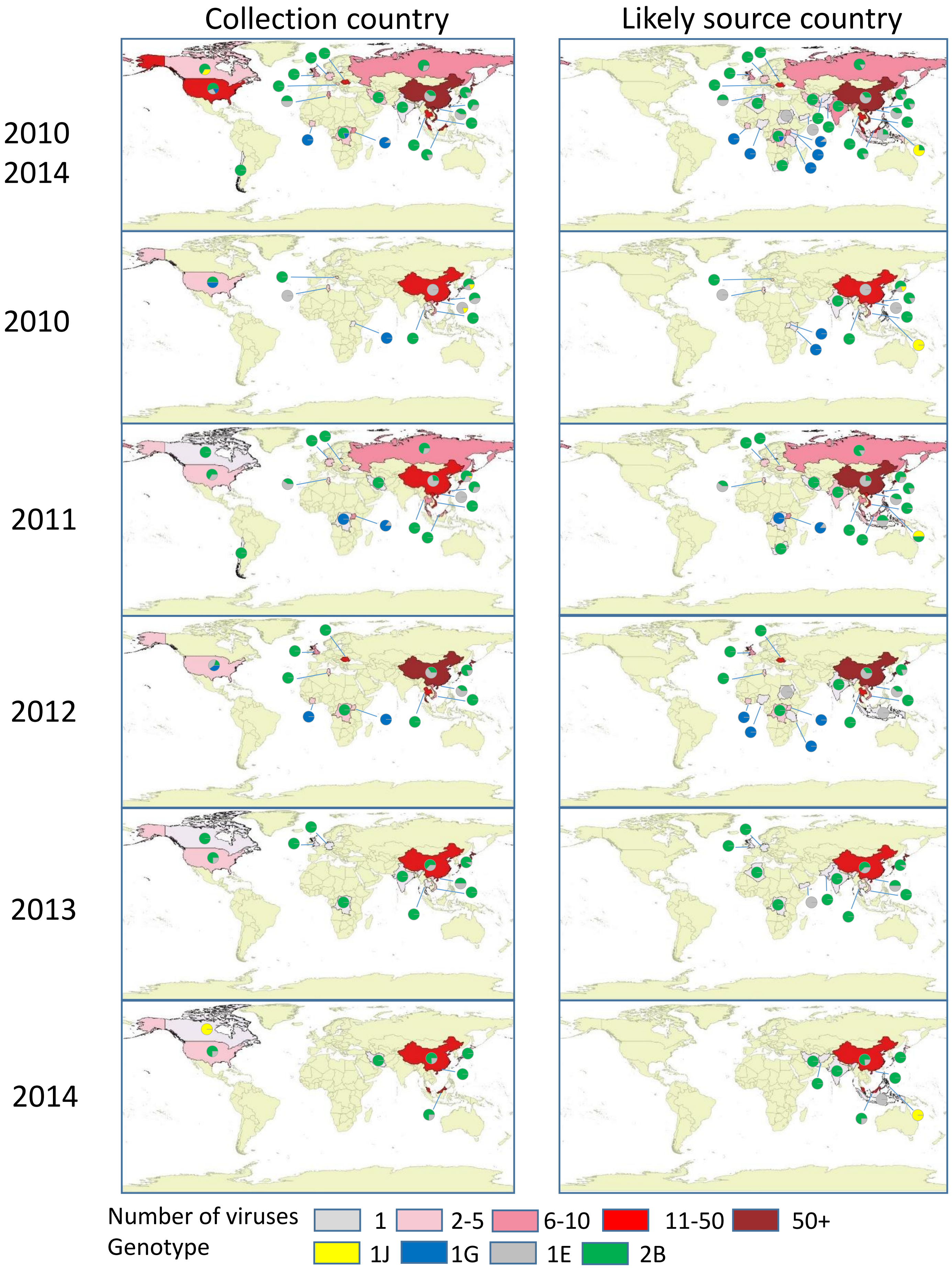

Supplementary Figure 1

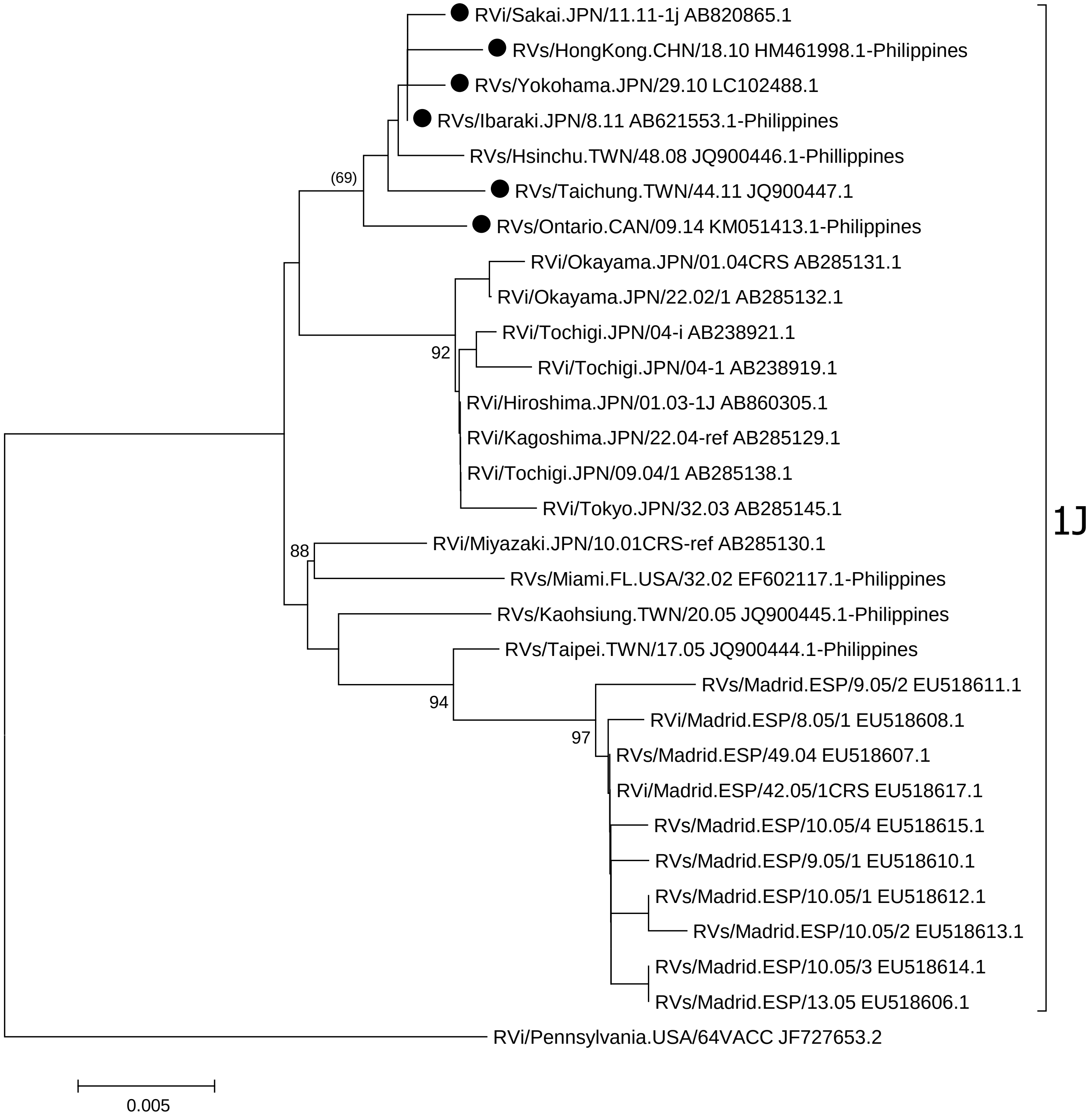

Supplementary Figure 2

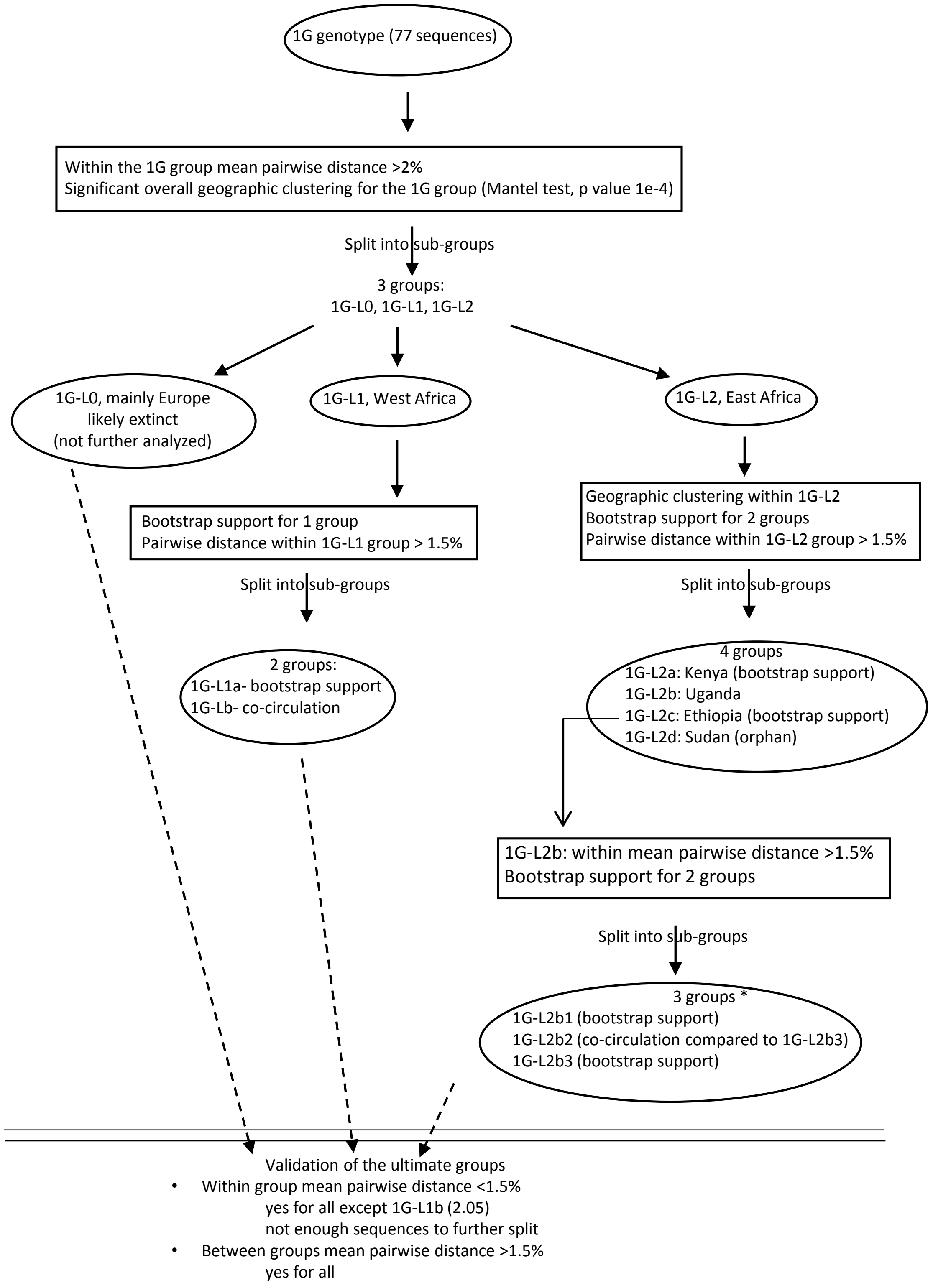

Supplementary Figure 3

Supplementary Figure 4

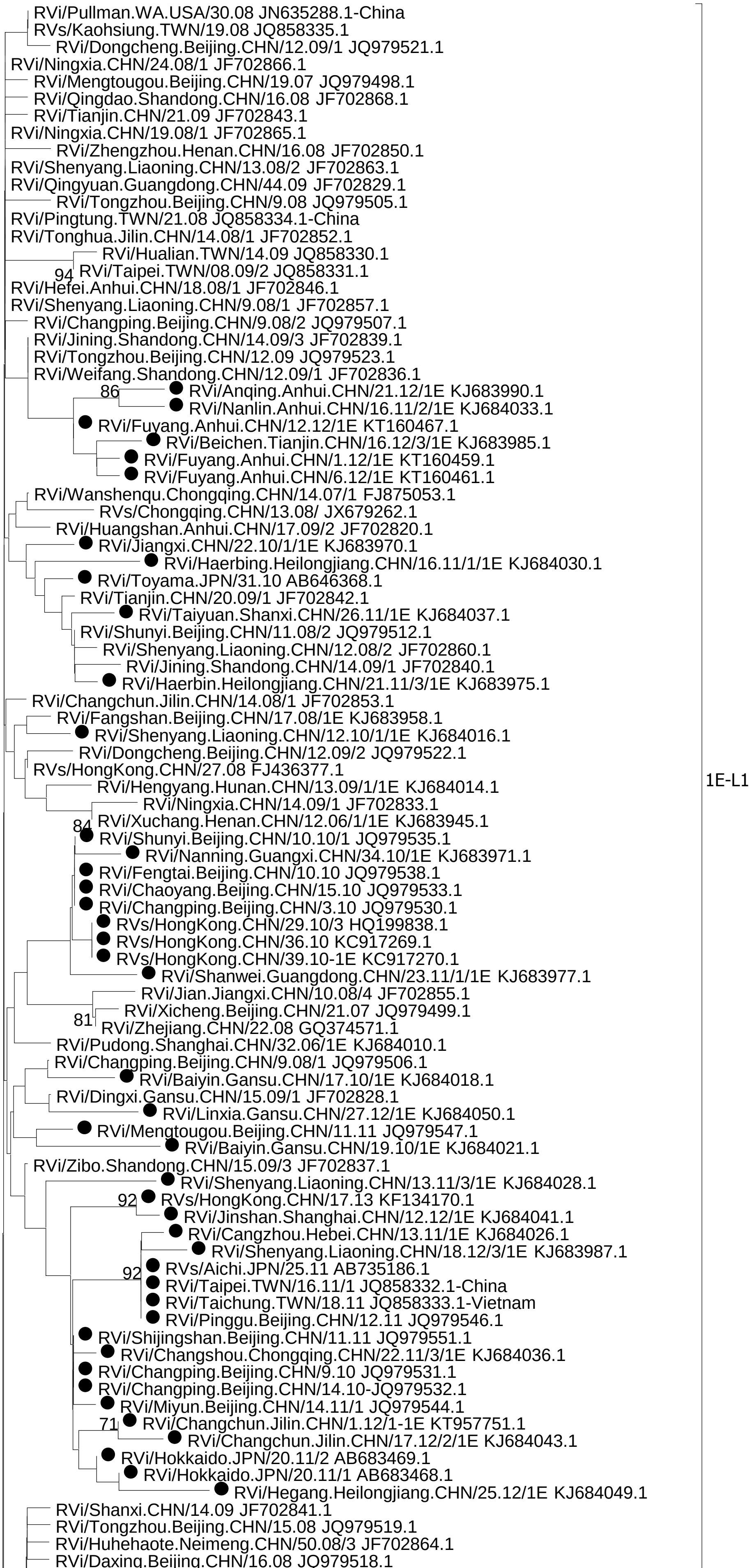

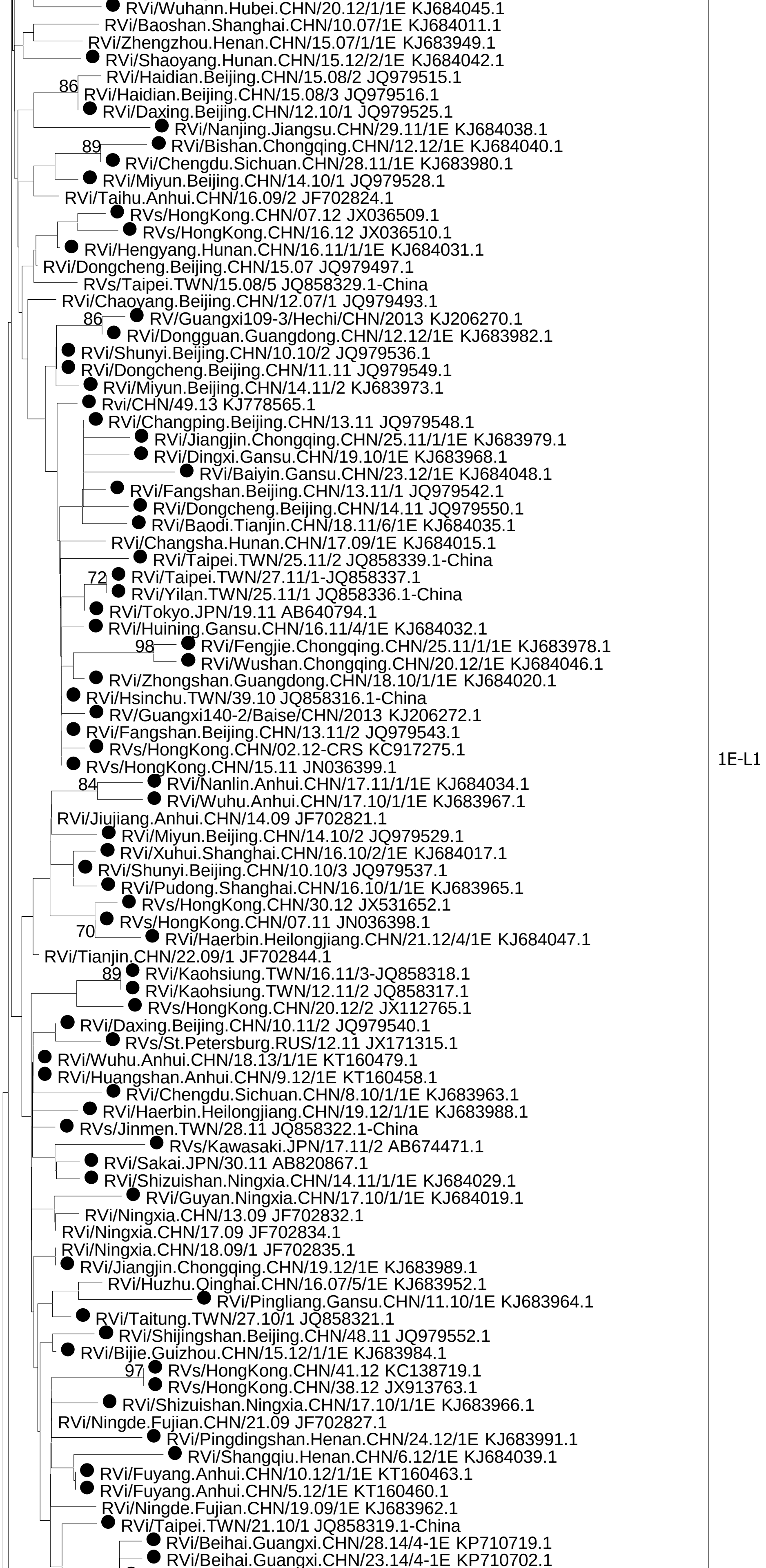
RVi/Wuhann.Hubei.CHN/20.12/1/1E KJ684045.1
RVi/Baoshan.Shanghai.CHN/10.07/1E KJ684011.1
RVi/Zhengzhou.Henan.CHN/15.07/1/1E KJ683949.1
RVi/Shaoyang.Hunan.CHN/15.12/2/1E KJ684042.1
RVi/Haidian.Beijing.CHN/15.08/2 JQ979515.1
86
RVi/Haidian.Beijing.CHN/15.08/3 JQ979516.1
RVi/Daxing.Beijing.CHN/12.10/1 JQ979525.1
RVi/Nanjing.Jiangsu.CHN/29.11/1E KJ684038.1
89
RVi/Bishan.Chongqing.CHN/12.12/1E KJ684040.1
RVi/Chengdu.Sichuan.CHN/28.11/1E KJ683980.1
RVi/Miyun.Beijing.CHN/14.10/1 JQ979528.1
RVi/Taihu.Anhui.CHN/16.09/2 JF702824.1
RVs/HongKong.CHN/07.12 JX036509.1
RVs/HongKong.CHN/16.12 JX036510.1
RVi/Hengyang.Hunan.CHN/16.11/1/1E KJ684031.1
RVi/Dongcheng.Beijing.CHN/15.07 JQ979497.1
RVs/Taipei.TWN/15.08/5 JQ858329.1-China
RVi/Chaoyang.Beijing.CHN/12.07/1 JQ979493.1
86
RV/Guangxi109-3/Hechi/CHN/2013 KJ206270.1
RVi/Dongguan.Guangdong.CHN/12.12/1E KJ683982.1
RVi/Shunyi.Beijing.CHN/10.10/2 JQ979536.1
RVi/Dongcheng.Beijing.CHN/11.11 JQ979549.1
RVi/Miyun.Beijing.CHN/14.11/2 KJ683973.1
Rvi/CHN/49.13 KJ778565.1
RVi/Changping.Beijing.CHN/13.11 JQ979548.1
RVi/Jiangjin.Chongqing.CHN/25.11/1/1E KJ683979.1
RVi/Dingxi.Gansu.CHN/19.10/1E KJ683968.1
RVi/Baiyin.Gansu.CHN/23.12/1E KJ684048.1
RVi/Fangshan.Beijing.CHN/13.11/1 JQ979542.1
RVi/Dongcheng.Beijing.CHN/14.11 JQ979550.1
RVi/Baodi.Tianjin.CHN/18.11/6/1E KJ684035.1
RVi/Changsha.Hunan.CHN/17.09/1E KJ684015.1
RVi/Taipei.TWN/25.11/2 JQ858339.1-China
72
RVi/Taipei.TWN/27.11/1-JQ858337.1
RVi/Yilan.TWN/25.11/1 JQ858336.1-China
RVi/Tokyo.JPN/19.11 AB640794.1
RVi/Huining.Gansu.CHN/16.11/4/1E KJ684032.1
98
RVi/Fengjie.Chongqing.CHN/25.11/1/1E KJ683978.1
RVi/Wushan.Chongqing.CHN/20.12/1E KJ684046.1
RVi/Zhongshan.Guangdong.CHN/18.10/1/1E KJ684020.1
RVi/Hsinchu.TWN/39.10 JQ858316.1-China
RV/Guangxi140-2/Baise/CHN/2013 KJ206272.1
RVi/Fangshan.Beijing.CHN/13.11/2 JQ979543.1
RVs/HongKong.CHN/02.12-CRS KC917275.1
RVs/HongKong.CHN/15.11 JN036399.1
1E-L1
84
RVi/Nanlin.Anhui.CHN/17.11/1/1E KJ684034.1
RVi/Wuhu.Anhui.CHN/17.10/1/1E KJ683967.1
RVi/Jiujiang.Anhui.CHN/14.09 JF702821.1
RVi/Miyun.Beijing.CHN/14.10/2 JQ979529.1
RVi/Xuhui.Shanghai.CHN/16.10/2/1E KJ684017.1
RVi/Shunyi.Beijing.CHN/10.10/3 JQ979537.1
RVi/Pudong.Shanghai.CHN/16.10/1/1E KJ683965.1
RVs/HongKong.CHN/30.12 JX531652.1
RVs/HongKong.CHN/07.11 JN036398.1
70
RVi/Haerbin.Heilongjiang.CHN/21.12/4/1E KJ684047.1
RVi/Tianjin.CHN/22.09/1 JF702844.1
89
RVi/Kaohsiung.TWN/16.11/3-JQ858318.1
RVi/Kaohsiung.TWN/12.11/2 JQ858317.1
RVs/HongKong.CHN/20.12/2 JX112765.1
RVi/Daxing.Beijing.CHN/10.11/2 JQ979540.1
RVs/St.Petersburg.RUS/12.11 JX171315.1
RVi/Wuhu.Anhui.CHN/18.13/1/1E KT160479.1
RVi/Huangshan.Anhui.CHN/9.12/1E KT160458.1
RVi/Chengdu.Sichuan.CHN/8.10/1/1E KJ683963.1
RVi/Haerbin.Heilongjiang.CHN/19.12/1/1E KJ683988.1
RVs/Jinmen.TWN/28.11 JQ858322.1-China
RVs/Kawasaki.JPN/17.11/2 AB674471.1
RVi/Sakai.JPN/30.11 AB820867.1
RVi/Shizuishan.Ningxia.CHN/14.11/1/1E KJ684029.1
RVi/Guyan.Ningxia.CHN/17.10/1/1E KJ684019.1
RVi/Ningxia.CHN/13.09 JF702832.1
RVi/Ningxia.CHN/17.09 JF702834.1
RVi/Ningxia.CHN/18.09/1 JF702835.1
RVi/Jiangjin.Chongqing.CHN/19.12/1E KJ683989.1
RVi/Huzhu.Qinghai.CHN/16.07/5/1E KJ683952.1
RVi/Pingliang.Gansu.CHN/11.10/1E KJ683964.1
RVi/Taitung.TWN/27.10/1 JQ858321.1
RVi/Shijingshan.Beijing.CHN/48.11 JQ979552.1
RVi/Bijie.Guizhou.CHN/15.12/1/1E KJ683984.1
97
RVs/HongKong.CHN/41.12 KC138719.1
RVs/HongKong.CHN/38.12 JX913763.1
RVi/Shizuishan.Ningxia.CHN/17.10/1/1E KJ683966.1
RVi/Ningde.Fujian.CHN/21.09 JF702827.1
RVi/Pingdingshan.Henan.CHN/24.12/1E KJ683991.1
RVi/Shangqiu.Henan.CHN/6.12/1E KJ684039.1
RVi/Fuyang.Anhui.CHN/10.12/1/1E KT160463.1
RVi/Fuyang.Anhui.CHN/5.12/1E KT160460.1
RVi/Ningde.Fujian.CHN/19.09/1E KJ683962.1
RVi/Taipei.TWN/21.10/1 JQ858319.1-China
RVi/Beihai.Guangxi.CHN/28.14/4-1E KP710719.1
RVi/Beihai.Guangxi.CHN/23.14/4-1E KP710702.1

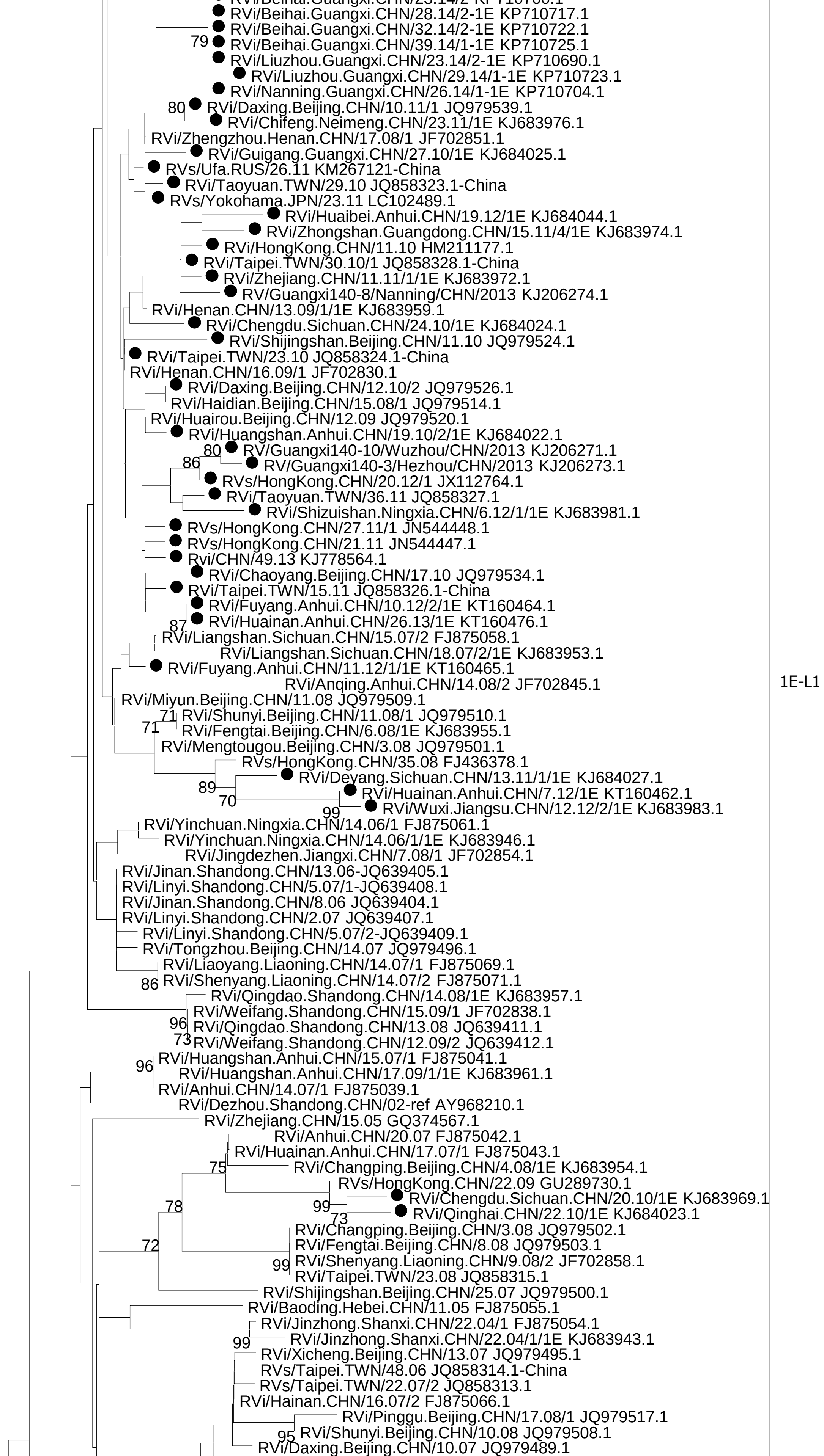
RVi/Beihai.Guangxi.CHN/28.14/2-1E KP710717.1
RVi/Beihai.Guangxi.CHN/32.14/2-1E KP710722.1
RVi/Beihai.Guangxi.CHN/39.14/1-1E KP710725.1
RVi/Liuzhou.Guangxi.CHN/23.14/2-1E KP710690.1
RVi/Liuzhou.Guangxi.CHN/29.14/1-1E KP710723.1
RVi/Nanning.Guangxi.CHN/26.14/1-1E KP710704.1
RVi/Daxing.Beijing.CHN/10.11/1 JQ979539.1
RVi/Chifeng.Neimeng.CHN/23.11/1E KJ683976.1
RVi/Zhengzhou.Henan.CHN/17.08/1 JF702851.1
RVi/Guigang.Guangxi.CHN/27.10/1E KJ684025.1
RVs/Ufa.RUS/26.11 KM267121-China
RVi/Taoyuan.TWN/29.10 JQ858323.1-China
RVs/Yokohama.JPN/23.11 LC102489.1
RVi/Huaibei.Anhui.CHN/19.12/1E KJ684044.1
RVi/Zhongshan.Guangdong.CHN/15.11/4/1E KJ683974.1
RVi/HongKong.CHN/11.10 HM211177.1
RVi/Taipei.TWN/30.10/1 JQ858328.1-China
RVi/Zhejiang.CHN/11.11/1/1E KJ683972.1
RV/Guangxi140-8/Nanning/CHN/2013 KJ206274.1
RVi/Henan.CHN/13.09/1/1E KJ683959.1
RVi/Chengdu.Sichuan.CHN/24.10/1E KJ684024.1
RVi/Shijingshan.Beijing.CHN/11.10 JQ979524.1
RVi/Taipei.TWN/23.10 JQ858324.1-China
RVi/Henan.CHN/16.09/1 JF702830.1
RVi/Daxing.Beijing.CHN/12.10/2 JQ979526.1
RVi/Haidian.Beijing.CHN/15.08/1 JQ979514.1
RVi/Huairou.Beijing.CHN/12.09 JQ979520.1
RVi/Huangshan.Anhui.CHN/19.10/2/1E KJ684022.1
RV/Guangxi140-10/Wuzhou/CHN/2013 KJ206271.1
RV/Guangxi140-3/Hezhou/CHN/2013 KJ206273.1
RVs/HongKong.CHN/20.12/1 JX112764.1
RVi/Taoyuan.TWN/36.11 JQ858327.1
RVi/Shizuishan.Ningxia.CHN/6.12/1/1E KJ683981.1
RVs/HongKong.CHN/27.11/1 JN544448.1
RVs/HongKong.CHN/21.11 JN544447.1
Rvi/CHN/49.13 KJ778564.1
RVi/Chaoyang.Beijing.CHN/17.10 JQ979534.1
RVi/Taipei.TWN/15.11 JQ858326.1-China
RVi/Fuyang.Anhui.CHN/10.12/2/1E KT160464.1
RVi/Huainan.Anhui.CHN/26.13/1E KT160476.1
RVi/Liangshan.Sichuan.CHN/15.07/2 FJ875058.1
RVi/Liangshan.Sichuan.CHN/18.07/2/1E KJ683953.1
RVi/Fuyang.Anhui.CHN/11.12/1/1E KT160465.1
RVi/Anqing.Anhui.CHN/14.08/2 JF702845.1
1E-L1
RVi/Miyun.Beijing.CHN/11.08 JQ979509.1
RVi/Shunyi.Beijing.CHN/11.08/1 JQ979510.1
RVi/Fengtai.Beijing.CHN/6.08/1E KJ683955.1
RVi/Mengtougou.Beijing.CHN/3.08 JQ979501.1
RVs/HongKong.CHN/35.08 FJ436378.1
RVi/Deyang.Sichuan.CHN/13.11/1/1E KJ684027.1
RVi/Huainan.Anhui.CHN/7.12/1E KT160462.1
RVi/Wuxi.Jiangsu.CHN/12.12/2/1E KJ683983.1
RVi/Yinchuan.Ningxia.CHN/14.06/1 FJ875061.1
RVi/Yinchuan.Ningxia.CHN/14.06/1/1E KJ683946.1
RVi/Jingdezhen.Jiangxi.CHN/7.08/1 JF702854.1
RVi/Jinan.Shandong.CHN/13.06-JQ639405.1
RVi/Linyi.Shandong.CHN/5.07/1-JQ639408.1
RVi/Jinan.Shandong.CHN/8.06 JQ639404.1
RVi/Linyi.Shandong.CHN/2.07 JQ639407.1
RVi/Linyi.Shandong.CHN/5.07/2-JQ639409.1
RVi/Tongzhou.Beijing.CHN/14.07 JQ979496.1
RVi/Liaoyang.Liaoning.CHN/14.07/1 FJ875069.1
RVi/Shenyang.Liaoning.CHN/14.07/2 FJ875071.1
RVi/Qingdao.Shandong.CHN/14.08/1E KJ683957.1
RVi/Weifang.Shandong.CHN/15.09/1 JF702838.1
RVi/Qingdao.Shandong.CHN/13.08 JQ639411.1
RVi/Weifang.Shandong.CHN/12.09/2 JQ639412.1
RVi/Huangshan.Anhui.CHN/15.07/1 FJ875041.1
RVi/Huangshan.Anhui.CHN/17.09/1/1E KJ683961.1
RVi/Anhui.CHN/14.07/1 FJ875039.1
RVi/Dezhou.Shandong.CHN/02-ref AY968210.1
RVi/Zhejiang.CHN/15.05 GQ374567.1
RVi/Anhui.CHN/20.07 FJ875042.1
RVi/Huainan.Anhui.CHN/17.07/1 FJ875043.1
RVi/Changping.Beijing.CHN/4.08/1E KJ683954.1
RVs/HongKong.CHN/22.09 GU289730.1
RVi/Chengdu.Sichuan.CHN/20.10/1E KJ683969.1
RVi/Qinghai.CHN/22.10/1E KJ684023.1
RVi/Changping.Beijing.CHN/3.08 JQ979502.1
RVi/Fengtai.Beijing.CHN/8.08 JQ979503.1
RVi/Shenyang.Liaoning.CHN/9.08/2 JF702858.1
RVi/Taipei.TWN/23.08 JQ858315.1
RVi/Shijingshan.Beijing.CHN/25.07 JQ979500.1
RVi/Baoding.Hebei.CHN/11.05 FJ875055.1
RVi/Jinzhong.Shanxi.CHN/22.04/1 FJ875054.1
RVi/Jinzhong.Shanxi.CHN/22.04/1/1E KJ683943.1
RVi/Xicheng.Beijing.CHN/13.07 JQ979495.1
RVs/Taipei.TWN/48.06 JQ858314.1-China
RVs/Taipei.TWN/22.07/2 JQ858313.1
RVi/Hainan.CHN/16.07/2 FJ875066.1
RVi/Pinggu.Beijing.CHN/17.08/1 JQ979517.1
RVi/Shunyi.Beijing.CHN/10.08 JQ979508.1
RVi/Daxing.Beijing.CHN/10.07 JQ979489.1

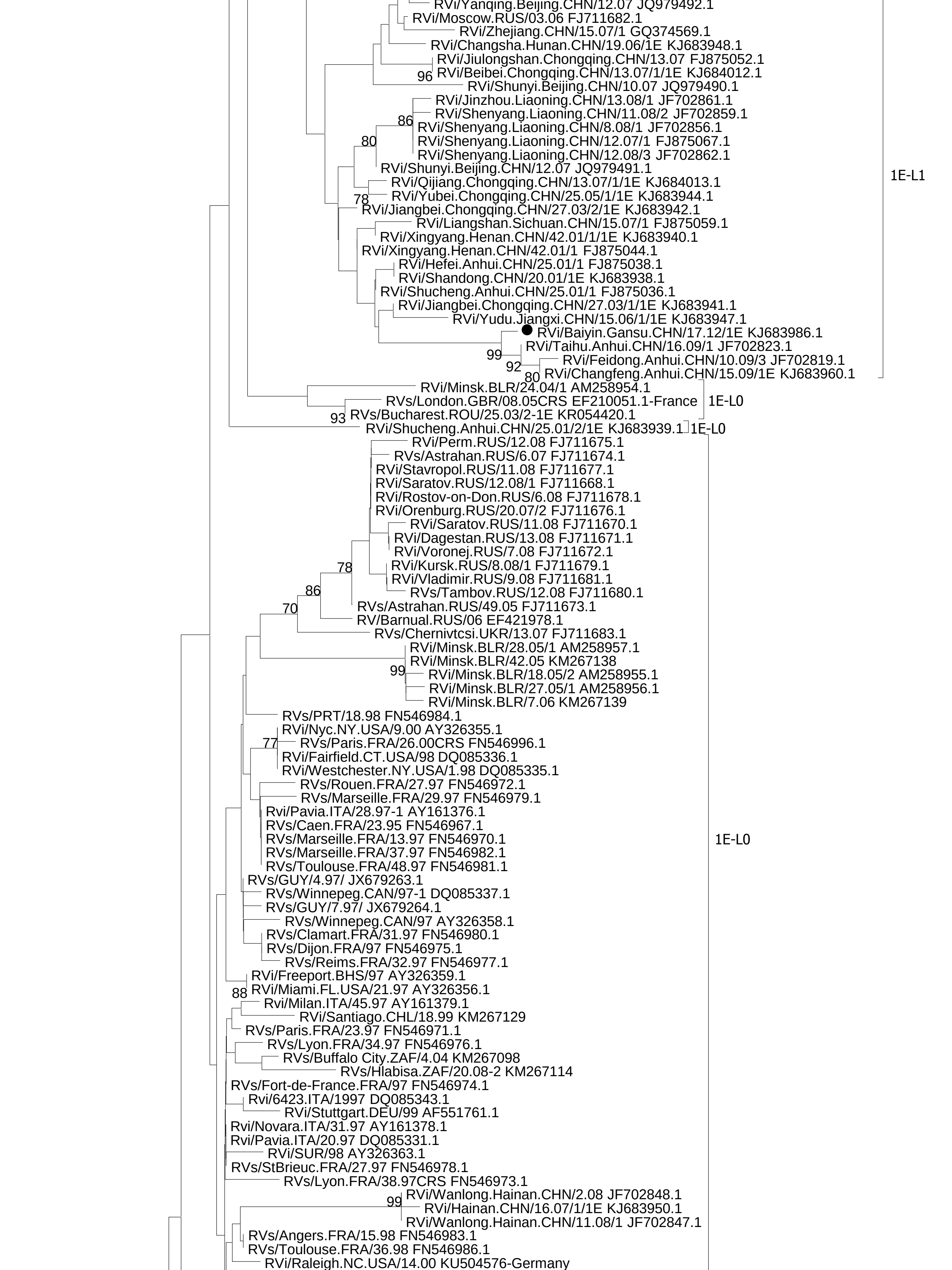
RVi/Yanqing.Beijing.CHN/12.07 JQ979492.1
RVi/Moscow.RUS/03.06 FJ711682.1
RVi/Zhejiang.CHN/15.07/1 GQ374569.1
RVi/Changsha.Hunan.CHN/19.06/1E KJ683948.1
RVi/Jiulongshan.Chongqing.CHN/13.07 FJ875052.1
96
RVi/Beibei.Chongqing.CHN/13.07/1/1E KJ684012.1
RVi/Shunyi.Beijing.CHN/10.07 JQ979490.1
RVi/Jinzhou.Liaoning.CHN/13.08/1 JF702861.1
RVi/Shenyang.Liaoning.CHN/11.08/2 JF702859.1
86
RVi/Shenyang.Liaoning.CHN/8.08/1 JF702856.1
80
RVi/Shenyang.Liaoning.CHN/12.07/1 FJ875067.1
RVi/Shenyang.Liaoning.CHN/12.08/3 JF702862.1
RVi/Shunyi.Beijing.CHN/12.07 JQ979491.1
RVi/Qijiang.Chongqing.CHN/13.07/1/1E KJ684013.1
1E-L1
RVi/Yubei.Chongqing.CHN/25.05/1/1E KJ683944.1
78
RVi/Jiangbei.Chongqing.CHN/27.03/2/1E KJ683942.1
RVi/Liangshan.Sichuan.CHN/15.07/1 FJ875059.1
RVi/Xingyang.Henan.CHN/42.01/1/1E KJ683940.1
RVi/Xingyang.Henan.CHN/42.01/1 FJ875044.1
RVi/Hefei.Anhui.CHN/25.01/1 FJ875038.1
RVi/Shandong.CHN/20.01/1E KJ683938.1
RVi/Shucheng.Anhui.CHN/25.01/1 FJ875036.1
RVi/Jiangbei.Chongqing.CHN/27.03/1/1E KJ683941.1
RVi/Yudu.Jiangxi.CHN/15.06/1/1E KJ683947.1
RVi/Baiyin.Gansu.CHN/17.12/1E KJ683986.1
99
RVi/Taihu.Anhui.CHN/16.09/1 JF702823.1
92
RVi/Feidong.Anhui.CHN/10.09/3 JF702819.1
80
RVi/Changfeng.Anhui.CHN/15.09/1E KJ683960.1
RVi/Minsk.BLR/24.04/1 AM258954.1
RVs/London.GBR/08.05CRS EF210051.1-France
1E-L0
93
RVs/Bucharest.ROU/25.03/2-1E KR054420.1
RVi/Shucheng.Anhui.CHN/25.01/2/1E KJ683939.1
1E-L0
RVi/Perm.RUS/12.08 FJ711675.1
RVs/Astrahan.RUS/6.07 FJ711674.1
RVi/Stavropol.RUS/11.08 FJ711677.1
RVi/Saratov.RUS/12.08/1 FJ711668.1
RVi/Rostov-on-Don.RUS/6.08 FJ711678.1
RVi/Orenburg.RUS/20.07/2 FJ711676.1
RVi/Saratov.RUS/11.08 FJ711670.1
RVi/Dagestan.RUS/13.08 FJ711671.1
RVi/Voronej.RUS/7.08 FJ711672.1
78
RVi/Kursk.RUS/8.08/1 FJ711679.1
RVi/Vladimir.RUS/9.08 FJ711681.1
86
RVs/Tambov.RUS/12.08 FJ711680.1
70
RVs/Astrahan.RUS/49.05 FJ711673.1
RV/Barnual.RUS/06 EF421978.1
RVs/Chernivtcsi.UKR/13.07 FJ711683.1
RVi/Minsk.BLR/28.05/1 AM258957.1
RVi/Minsk.BLR/42.05 KM267138
99
RVi/Minsk.BLR/18.05/2 AM258955.1
RVi/Minsk.BLR/27.05/1 AM258956.1
RVi/Minsk.BLR/7.06 KM267139
RVs/PRT/18.98 FN546984.1
RVi/Nyc.NY.USA/9.00 AY326355.1
77
RVs/Paris.FRA/26.00CRS FN546996.1
RVi/Fairfield.CT.USA/98 DQ085336.1
RVi/Westchester.NY.USA/1.98 DQ085335.1
RVs/Rouen.FRA/27.97 FN546972.1
RVs/Marseille.FRA/29.97 FN546979.1
Rvi/Pavia.ITA/28.97-1 AY161376.1
RVs/Caen.FRA/23.95 FN546967.1
RVs/Marseille.FRA/13.97 FN546970.1
1E-L0
RVs/Marseille.FRA/37.97 FN546982.1
RVs/Toulouse.FRA/48.97 FN546981.1
RVs/GUY/4.97/ JX679263.1
RVs/Winnepeg.CAN/97-1 DQ085337.1
RVs/GUY/7.97/ JX679264.1
RVs/Winnepeg.CAN/97 AY326358.1
RVs/Clamart.FRA/31.97 FN546980.1
RVs/Dijon.FRA/97 FN546975.1
RVs/Reims.FRA/32.97 FN546977.1
RVi/Freeport.BHS/97 AY326359.1
88
RVi/Miami.FL.USA/21.97 AY326356.1
Rvi/Milan.ITA/45.97 AY161379.1
RVi/Santiago.CHL/18.99 KM267129
RVs/Paris.FRA/23.97 FN546971.1
RVs/Lyon.FRA/34.97 FN546976.1
RVs/Buffalo City.ZAF/4.04 KM267098
RVs/Hlabisa.ZAF/20.08-2 KM267114
RVs/Fort-de-France.FRA/97 FN546974.1
Rvi/6423.ITA/1997 DQ085343.1
RVi/Stuttgart.DEU/99 AF551761.1
Rvi/Novara.ITA/31.97 AY161378.1
Rvi/Pavia.ITA/20.97 DQ085331.1
RVi/SUR/98 AY326363.1
RVs/StBrieuc.FRA/27.97 FN546978.1
RVs/Lyon.FRA/38.97CRS FN546973.1
RVi/Wanlong.Hainan.CHN/2.08 JF702848.1
99
RVi/Hainan.CHN/16.07/1/1E KJ683950.1
RVi/Wanlong.Hainan.CHN/11.08/1 JF702847.1
RVs/Angers.FRA/15.98 FN546983.1
RVs/Toulouse.FRA/36.98 FN546986.1
RVi/Raleigh.NC.USA/14.00 KU504576-Germany

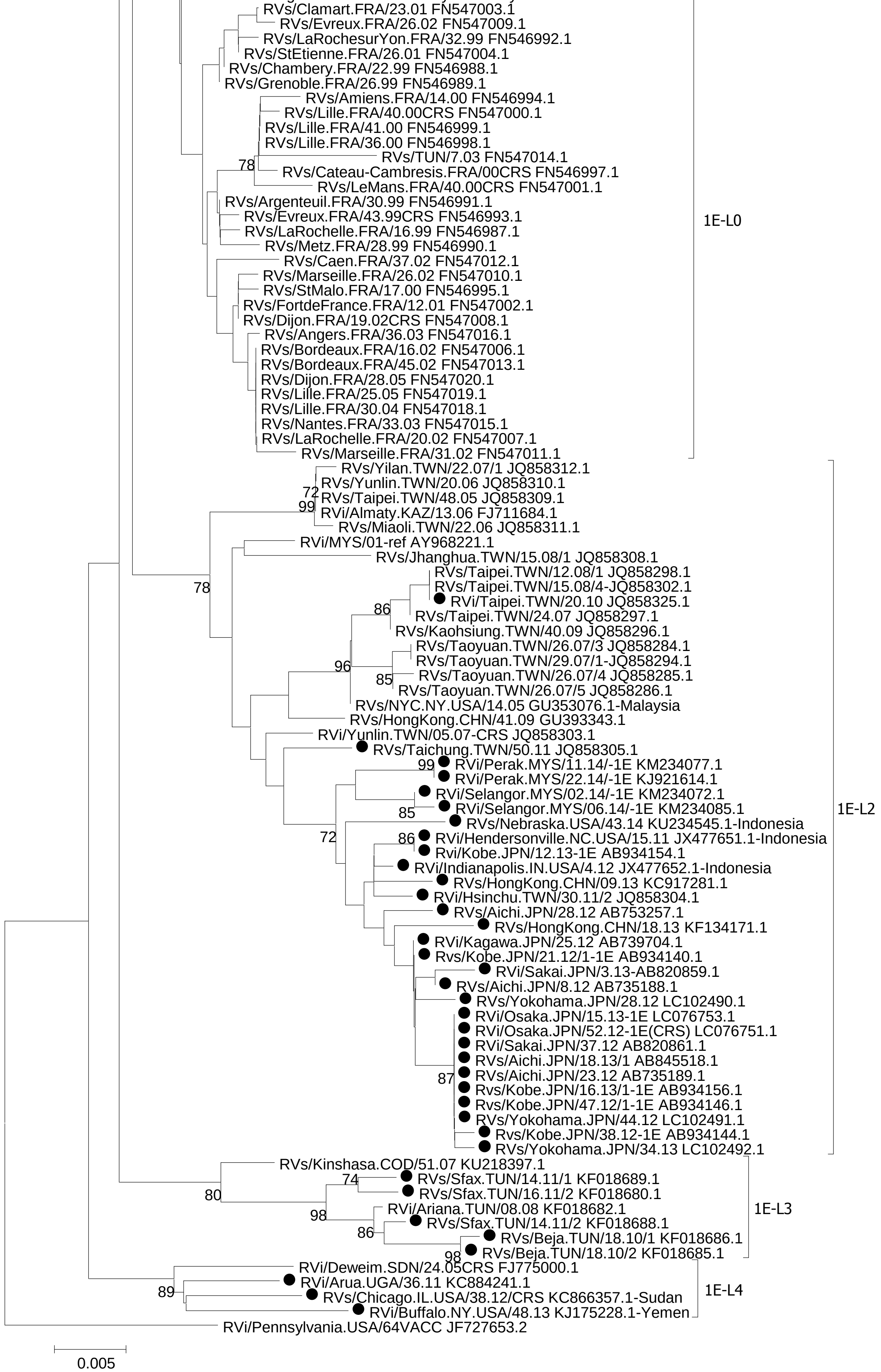
RVs/Clamart.FRA/23.01 FN547003.1
RVs/Evreux.FRA/26.02 FN547009.1
RVs/LaRochesurYon.FRA/32.99 FN546992.1
RVs/StEtienne.FRA/26.01 FN547004.1
RVs/Chambery.FRA/22.99 FN546988.1
RVs/Grenoble.FRA/26.99 FN546989.1
RVs/Amiens.FRA/14.00 FN546994.1
RVs/Lille.FRA/40.00CRS FN547000.1
RVs/Lille.FRA/41.00 FN546999.1
RVs/Lille.FRA/36.00 FN546998.1
RVs/TUN/7.03 FN547014.1
78
RVs/Cateau-Cambresis.FRA/00CRS FN546997.1
RVs/LeMans.FRA/40.00CRS FN547001.1
RVs/Argenteuil.FRA/30.99 FN546991.1
RVs/Evreux.FRA/43.99CRS FN546993.1
RVs/LaRochelle.FRA/16.99 FN546987.1
RVs/Metz.FRA/28.99 FN546990.1
RVs/Caen.FRA/37.02 FN547012.1
RVs/Marseille.FRA/26.02 FN547010.1
RVs/StMalo.FRA/17.00 FN546995.1
RVs/FortdeFrance.FRA/12.01 FN547002.1
RVs/Dijon.FRA/19.02CRS FN547008.1
RVs/Angers.FRA/36.03 FN547016.1
RVs/Bordeaux.FRA/16.02 FN547006.1
RVs/Bordeaux.FRA/45.02 FN547013.1
RVs/Dijon.FRA/28.05 FN547020.1
RVs/Lille.FRA/25.05 FN547019.1
RVs/Lille.FRA/30.04 FN547018.1
RVs/Nantes.FRA/33.03 FN547015.1
RVs/LaRochelle.FRA/20.02 FN547007.1
RVs/Marseille.FRA/31.02 FN547011.1
1E-L0
RVs/Yilan.TWN/22.07/1 JQ858312.1
RVs/Yunlin.TWN/20.06 JQ858310.1
72
RVs/Taipei.TWN/48.05 JQ858309.1
99
RVi/Almaty.KAZ/13.06 FJ711684.1
RVs/Miaoli.TWN/22.06 JQ858311.1
RVi/MYS/01-ref AY968221.1
RVs/Jhanghua.TWN/15.08/1 JQ858308.1
78
RVs/Taipei.TWN/12.08/1 JQ858298.1
RVs/Taipei.TWN/15.08/4-JQ858302.1
RVi/Taipei.TWN/20.10 JQ858325.1
86
RVs/Taipei.TWN/24.07 JQ858297.1
RVs/Kaohsiung.TWN/40.09 JQ858296.1
RVs/Taoyuan.TWN/26.07/3 JQ858284.1
RVs/Taoyuan.TWN/29.07/1-JQ858294.1
96
RVs/Taoyuan.TWN/26.07/4 JQ858285.1
85
RVs/Taoyuan.TWN/26.07/5 JQ858286.1
RVs/NYC.NY.USA/14.05 GU353076.1-Malaysia
RVs/HongKong.CHN/41.09 GU393343.1
RVi/Yunlin.TWN/05.07-CRS JQ858303.1
RVs/Taichung.TWN/50.11 JQ858305.1
99
RVi/Perak.MYS/11.14/-1E KM234077.1
RVi/Perak.MYS/22.14/-1E KJ921614.1
RVi/Selangor.MYS/02.14/-1E KM234072.1
85
RVi/Selangor.MYS/06.14/-1E KM234085.1
RVs/Nebraska.USA/43.14 KU234545.1-Indonesia
72
86
RVi/Hendersonville.NC.USA/15.11 JX477651.1-Indonesia
Rvi/Kobe.JPN/12.13-1E AB934154.1
RVi/Indianapolis.IN.USA/4.12 JX477652.1-Indonesia
RVs/HongKong.CHN/09.13 KC917281.1
RVi/Hsinchu.TWN/30.11/2 JQ858304.1
RVs/Aichi.JPN/28.12 AB753257.1
RVs/HongKong.CHN/18.13 KF134171.1
RVi/Kagawa.JPN/25.12 AB739704.1
Rvs/Kobe.JPN/21.12/1-1E AB934140.1
RVi/Sakai.JPN/3.13-AB820859.1
RVs/Aichi.JPN/8.12 AB735188.1
RVs/Yokohama.JPN/28.12 LC102490.1
RVi/Osaka.JPN/15.13-1E LC076753.1
RVi/Osaka.JPN/52.12-1E(CRS) LC076751.1
RVi/Sakai.JPN/37.12 AB820861.1
RVs/Aichi.JPN/18.13/1 AB845518.1
RVs/Aichi.JPN/23.12 AB735189.1
87
Rvs/Kobe.JPN/16.13/1-1E AB934156.1
Rvs/Kobe.JPN/47.12/1-1E AB934146.1
RVs/Yokohama.JPN/44.12 LC102491.1
Rvs/Kobe.JPN/38.12-1E AB934144.1
RVs/Yokohama.JPN/34.13 LC102492.1
1E-L2
RVs/Kinshasa.COD/51.07 KU218397.1
74
RVs/Sfax.TUN/14.11/1 KF018689.1
80
RVs/Sfax.TUN/16.11/2 KF018680.1
RVi/Ariana.TUN/08.08 KF018682.1
98
RVs/Sfax.TUN/14.11/2 KF018688.1
86
RVs/Beja.TUN/18.10/1 KF018686.1
98
RVs/Beja.TUN/18.10/2 KF018685.1
1E-L3
RVi/Deweim.SDN/24.05CRS FJ775000.1
RVi/Arua.UGA/36.11 KC884241.1
89
RVs/Chicago.IL.USA/38.12/CRS KC866357.1-Sudan
RVi/Buffalo.NY.USA/48.13 KJ175228.1-Yemen
1E-L4
RVi/Pennsylvania.USA/64VACC JF727653.2
0.005

Supplementary Figure 5

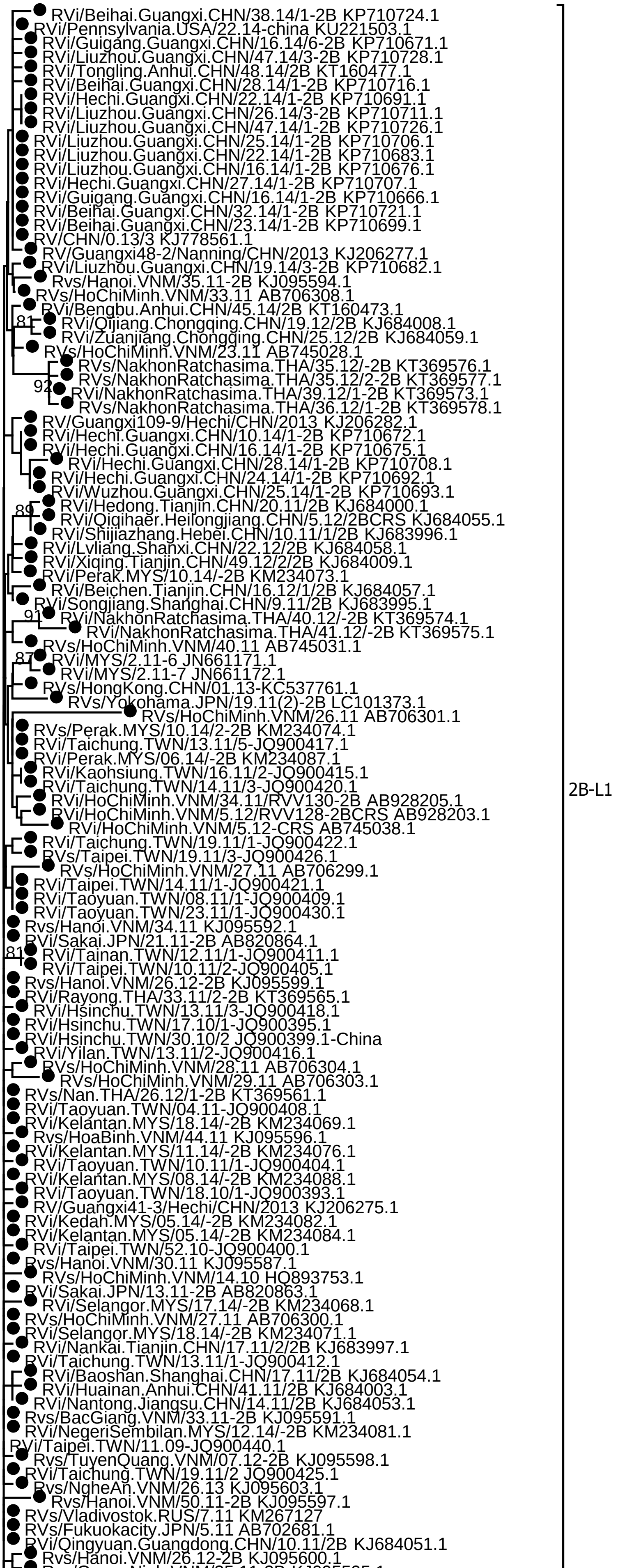

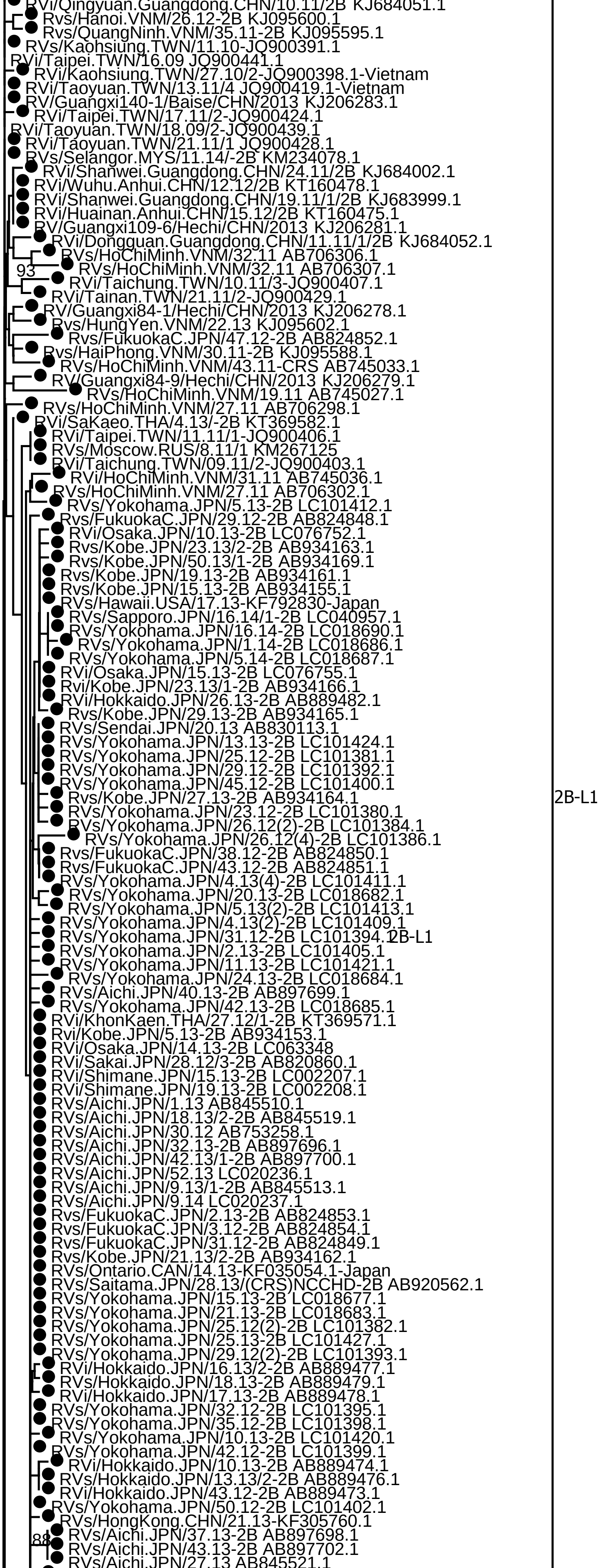
RVi/Qingyuan.Guangdong.CHN/10.11/2B KJ684051.1
Rvs/Hanoi.VNM/26.12-2B KJ095600.1
Rvs/QuangNinh.VNM/35.11-2B KJ095595.1
RVs/Kaohsiung.TWN/11.10-JQ900391.1
RVi/Taipei.TWN/16.09 JQ900441.1
RVi/Kaohsiung.TWN/27.10/2-JQ900398.1-Vietnam
RVi/Taoyuan.TWN/13.11/4 JQ900419.1-Vietnam
RV/Guangxi140-1/Baise/CHN/2013 KJ206283.1
RVi/Taipei.TWN/17.11/2-JQ900424.1
RVi/Taoyuan.TWN/18.09/2-JQ900439.1
RVi/Taoyuan.TWN/21.11/1 JQ900428.1
RVs/Selangor.MYS/11.14/-2B KM234078.1
RVi/Shanwei.Guangdong.CHN/24.11/2B KJ684002.1
RVi/Wuhu.Anhui.CHN/12.12/2B KT160478.1
RVi/Shanwei.Guangdong.CHN/19.11/1/2B KJ683999.1
RVi/Huainan.Anhui.CHN/15.12/2B KT160475.1
RV/Guangxi109-6/Hechi/CHN/2013 KJ206281.1
RVi/Dongguan.Guangdong.CHN/11.11/1/2B KJ684052.1
RVs/HoChiMinh.VNM/32.11 AB706306.1
93
RVs/HoChiMinh.VNM/32.11 AB706307.1
RVi/Taichung.TWN/10.11/3-JQ900407.1
RVi/Tainan.TWN/21.11/2-JQ900429.1
RV/Guangxi84-1/Hechi/CHN/2013 KJ206278.1
Rvs/HungYen.VNM/22.13 KJ095602.1
Rvs/FukuokaC.JPN/47.12-2B AB824852.1
Rvs/HaiPhong.VNM/30.11-2B KJ095588.1
RVs/HoChiMinh.VNM/43.11-CRS AB745033.1
RV/Guangxi84-9/Hechi/CHN/2013 KJ206279.1
RVs/HoChiMinh.VNM/19.11 AB745027.1
RVs/HoChiMinh.VNM/27.11 AB706298.1
RVi/SaKaeo.THA/4.13/-2B KT369582.1
RVi/Taipei.TWN/11.11/1-JQ900406.1
RVs/Moscow.RUS/8.11/1 KM267125
RVi/Taichung.TWN/09.11/2-JQ900403.1
RVi/HoChiMinh.VNM/31.11 AB745036.1
RVs/HoChiMinh.VNM/27.11 AB706302.1
RVs/Yokohama.JPN/5.13-2B LC101412.1
Rvs/FukuokaC.JPN/29.12-2B AB824848.1
RVi/Osaka.JPN/10.13-2B LC076752.1
Rvs/Kobe.JPN/23.13/2-2B AB934163.1
Rvs/Kobe.JPN/50.13/1-2B AB934169.1
Rvs/Kobe.JPN/19.13-2B AB934161.1
Rvs/Kobe.JPN/15.13-2B AB934155.1
RVs/Hawaii.USA/17.13-KF792830-Japan
RVs/Sapporo.JPN/16.14/1-2B LC040957.1
RVs/Yokohama.JPN/16.14-2B LC018690.1
RVs/Yokohama.JPN/1.14-2B LC018686.1
RVs/Yokohama.JPN/5.14-2B LC018687.1
RVi/Osaka.JPN/15.13-2B LC076755.1
Rvi/Kobe.JPN/23.13/1-2B AB934166.1
RVi/Hokkaido.JPN/26.13-2B AB889482.1
Rvs/Kobe.JPN/29.13-2B AB934165.1
RVs/Sendai.JPN/20.13 AB830113.1
RVs/Yokohama.JPN/13.13-2B LC101424.1
RVs/Yokohama.JPN/25.12-2B LC101381.1
RVs/Yokohama.JPN/29.12-2B LC101392.1
RVs/Yokohama.JPN/45.12-2B LC101400.1
Rvs/Kobe.JPN/27.13-2B AB934164.1
RVs/Yokohama.JPN/23.12-2B LC101380.1
RVs/Yokohama.JPN/26.12(2)-2B LC101384.1
RVs/Yokohama.JPN/26.12(4)-2B LC101386.1
Rvs/FukuokaC.JPN/38.12-2B AB824850.1
Rvs/FukuokaC.JPN/43.12-2B AB824851.1
RVs/Yokohama.JPN/4.13(4)-2B LC101411.1
RVs/Yokohama.JPN/20.13-2B LC018682.1
RVs/Yokohama.JPN/5.13(2)-2B LC101413.1
RVs/Yokohama.JPN/4.13(2)-2B LC101409.1
RVs/Yokohama.JPN/31.12-2B LC101394.1
2B-L1
RVs/Yokohama.JPN/2.13-2B LC101405.1
RVs/Yokohama.JPN/11.13-2B LC101421.1
RVs/Yokohama.JPN/24.13-2B LC018684.1
RVs/Aichi.JPN/40.13-2B AB897699.1
RVs/Yokohama.JPN/42.13-2B LC018685.1
RVi/KhonKaen.THA/27.12/1-2B KT369571.1
Rvi/Kobe.JPN/5.13-2B AB934153.1
RVi/Osaka.JPN/14.13-2B LC063348
RVi/Sakai.JPN/28.12/3-2B AB820860.1
RVi/Shimane.JPN/15.13-2B LC002207.1
RVi/Shimane.JPN/19.13-2B LC002208.1
RVs/Aichi.JPN/1.13 AB845510.1
RVs/Aichi.JPN/18.13/2-2B AB845519.1
RVs/Aichi.JPN/30.12 AB753258.1
RVs/Aichi.JPN/32.13-2B AB897696.1
RVs/Aichi.JPN/42.13/1-2B AB897700.1
RVs/Aichi.JPN/52.13 LC020236.1
RVs/Aichi.JPN/9.13/1-2B AB845513.1
RVs/Aichi.JPN/9.14 LC020237.1
Rvs/FukuokaC.JPN/2.13-2B AB824853.1
Rvs/FukuokaC.JPN/3.12-2B AB824854.1
Rvs/FukuokaC.JPN/31.12-2B AB824849.1
Rvs/Kobe.JPN/21.13/2-2B AB934162.1
RVs/Ontario.CAN/14.13-KF035054.1-Japan
RVs/Saitama.JPN/28.13/(CRS)NCCHD-2B AB920562.1
RVs/Yokohama.JPN/15.13-2B LC018677.1
RVs/Yokohama.JPN/21.13-2B LC018683.1
RVs/Yokohama.JPN/25.12(2)-2B LC101382.1
RVs/Yokohama.JPN/25.13-2B LC101427.1
RVs/Yokohama.JPN/29.12(2)-2B LC101393.1
RVi/Hokkaido.JPN/16.13/2-2B AB889477.1
RVs/Hokkaido.JPN/18.13-2B AB889479.1
RVi/Hokkaido.JPN/17.13-2B AB889478.1
RVs/Yokohama.JPN/32.12-2B LC101395.1
RVs/Yokohama.JPN/35.12-2B LC101398.1
RVs/Yokohama.JPN/10.13-2B LC101420.1
RVs/Yokohama.JPN/42.12-2B LC101399.1
RVi/Hokkaido.JPN/10.13-2B AB889474.1
RVs/Hokkaido.JPN/13.13/2-2B AB889476.1
RVi/Hokkaido.JPN/43.12-2B AB889473.1
RVs/Yokohama.JPN/50.12-2B LC101402.1
RVs/HongKong.CHN/21.13-KF305760.1
88
RVs/Aichi.JPN/37.13-2B AB897698.1
RVs/Aichi.JPN/43.13-2B AB897702.1
RVs/Aichi.JPN/27.13 AB845521.1
2B-L1

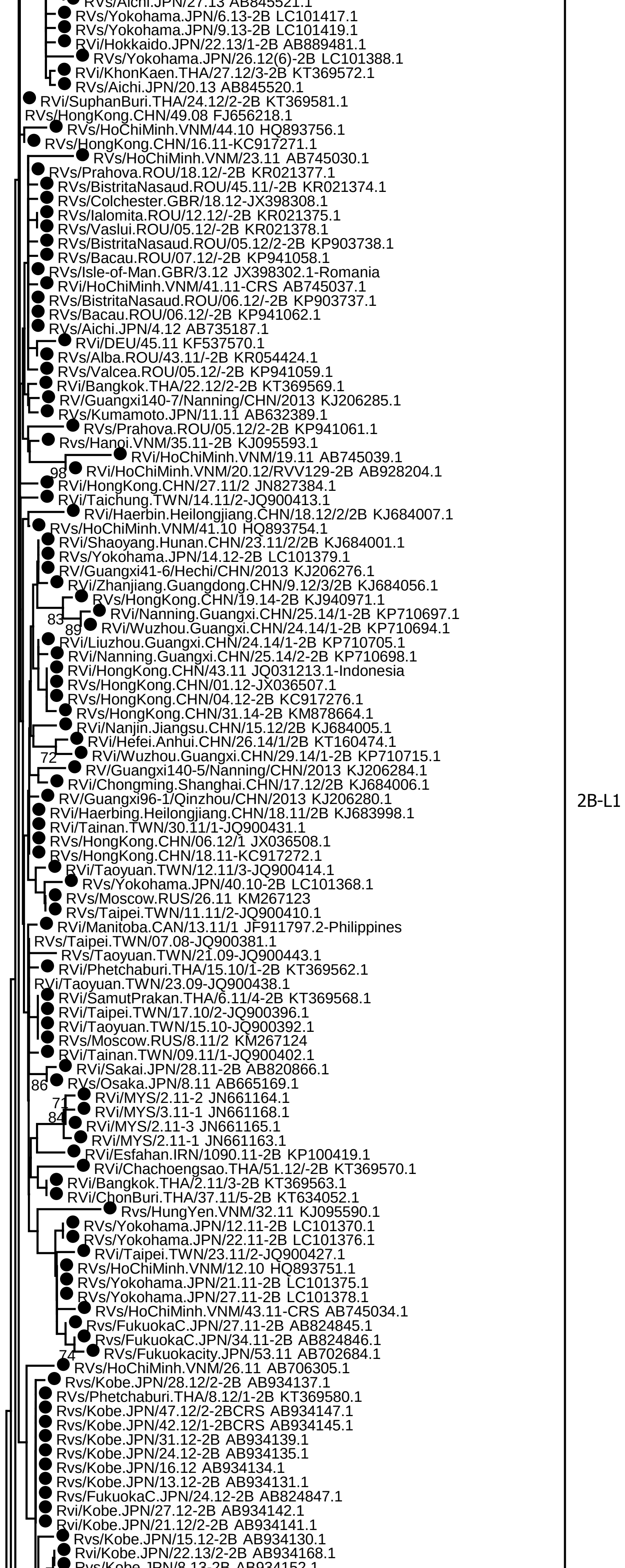
RVs/Aichi.JPN/27.13 AB845521.1
RVs/Yokohama.JPN/6.13-2B LC101417.1
RVs/Yokohama.JPN/9.13-2B LC101419.1
RVi/Hokkaido.JPN/22.13/1-2B AB889481.1
RVs/Yokohama.JPN/26.12(6)-2B LC101388.1
RVi/KhonKaen.THA/27.12/3-2B KT369572.1
RVs/Aichi.JPN/20.13 AB845520.1
RVi/SuphanBuri.THA/24.12/2-2B KT369581.1
RVs/HongKong.CHN/49.08 FJ656218.1
RVs/HoChiMinh.VNM/44.10 HQ893756.1
RVs/HongKong.CHN/16.11-KC917271.1
RVs/HoChiMinh.VNM/23.11 AB745030.1
RVs/Prahova.ROU/18.12/-2B KR021377.1
RVs/BistritaNasaud.ROU/45.11/-2B KR021374.1
RVs/Colchester.GBR/18.12-JX398308.1
RVs/Ialomita.ROU/12.12/-2B KR021375.1
RVs/Vaslui.ROU/05.12/-2B KR021378.1
RVs/BistritaNasaud.ROU/05.12/2-2B KP903738.1
RVs/Bacau.ROU/07.12/-2B KP941058.1
RVs/Isle-of-Man.GBR/3.12 JX398302.1-Romania
RVi/HoChiMinh.VNM/41.11-CRS AB745037.1
RVs/BistritaNasaud.ROU/06.12/-2B KP903737.1
RVs/Bacau.ROU/06.12/-2B KP941062.1
RVs/Aichi.JPN/4.12 AB735187.1
RVi/DEU/45.11 KF537570.1
RVs/Alba.ROU/43.11/-2B KR054424.1
RVs/Valcea.ROU/05.12/-2B KP941059.1
RVi/Bangkok.THA/22.12/2-2B KT369569.1
RV/Guangxi140-7/Nanning/CHN/2013 KJ206285.1
RVs/Kumamoto.JPN/11.11 AB632389.1
RVs/Prahova.ROU/05.12/2-2B KP941061.1
Rvs/Hanoi.VNM/35.11-2B KJ095593.1
RVi/HoChiMinh.VNM/19.11 AB745039.1
98
RVi/HoChiMinh.VNM/20.12/RVV129-2B AB928204.1
RVi/HongKong.CHN/27.11/2 JN827384.1
RVi/Taichung.TWN/14.11/2-JQ900413.1
RVi/Haerbin.Heilongjiang.CHN/18.12/2/2B KJ684007.1
RVs/HoChiMinh.VNM/41.10 HQ893754.1
RVi/Shaoyang.Hunan.CHN/23.11/2/2B KJ684001.1
RVs/Yokohama.JPN/14.12-2B LC101379.1
RV/Guangxi41-6/Hechi/CHN/2013 KJ206276.1
RVi/Zhanjiang.Guangdong.CHN/9.12/3/2B KJ684056.1
RVs/HongKong.CHN/19.14-2B KJ940971.1
83
RVi/Nanning.Guangxi.CHN/25.14/1-2B KP710697.1
89
RVi/Wuzhou.Guangxi.CHN/24.14/1-2B KP710694.1
RVi/Liuzhou.Guangxi.CHN/24.14/1-2B KP710705.1
RVi/Nanning.Guangxi.CHN/25.14/2-2B KP710698.1
RVi/HongKong.CHN/43.11 JQ031213.1-Indonesia
RVs/HongKong.CHN/01.12-JX036507.1
RVs/HongKong.CHN/04.12-2B KC917276.1
RVs/HongKong.CHN/31.14-2B KM878664.1
RVi/Nanjin.Jiangsu.CHN/15.12/2B KJ684005.1
RVi/Hefei.Anhui.CHN/26.14/1/2B KT160474.1
72
RVi/Wuzhou.Guangxi.CHN/29.14/1-2B KP710715.1
RV/Guangxi140-5/Nanning/CHN/2013 KJ206284.1
RVi/Chongming.Shanghai.CHN/17.12/2B KJ684006.1
RV/Guangxi96-1/Qinzhou/CHN/2013 KJ206280.1
RVi/Haerbing.Heilongjiang.CHN/18.11/2B KJ683998.1
RVi/Tainan.TWN/30.11/1-JQ900431.1
RVs/HongKong.CHN/06.12/1 JX036508.1
RVs/HongKong.CHN/18.11-KC917272.1
RVi/Taoyuan.TWN/12.11/3-JQ900414.1
RVs/Yokohama.JPN/40.10-2B LC101368.1
RVs/Moscow.RUS/26.11 KM267123
RVs/Taipei.TWN/11.11/2-JQ900410.1
RVi/Manitoba.CAN/13.11/1 JF911797.2-Philippines
RVs/Taipei.TWN/07.08-JQ900381.1
RVs/Taoyuan.TWN/21.09-JQ900443.1
RVi/Phetchaburi.THA/15.10/1-2B KT369562.1
RVi/Taoyuan.TWN/23.09-JQ900438.1
RVi/SamutPrakan.THA/6.11/4-2B KT369568.1
RVi/Taipei.TWN/17.10/2-JQ900396.1
RVi/Taoyuan.TWN/15.10-JQ900392.1
RVs/Moscow.RUS/8.11/2 KM267124
RVi/Tainan.TWN/09.11/1-JQ900402.1
RVi/Sakai.JPN/28.11-2B AB820866.1
86
RVs/Osaka.JPN/8.11 AB665169.1
71
RVi/MYS/2.11-2 JN661164.1
RVi/MYS/3.11-1 JN661168.1
84
RVi/MYS/2.11-3 JN661165.1
RVi/MYS/2.11-1 JN661163.1
RVi/Esfahan.IRN/1090.11-2B KP100419.1
RVi/Chachoengsao.THA/51.12/-2B KT369570.1
RVi/Bangkok.THA/2.11/3-2B KT369563.1
RVi/ChonBuri.THA/37.11/5-2B KT634052.1
Rvs/HungYen.VNM/32.11 KJ095590.1
RVs/Yokohama.JPN/12.11-2B LC101370.1
RVs/Yokohama.JPN/22.11-2B LC101376.1
RVi/Taipei.TWN/23.11/2-JQ900427.1
RVs/HoChiMinh.VNM/12.10 HQ893751.1
RVs/Yokohama.JPN/21.11-2B LC101375.1
RVs/Yokohama.JPN/27.11-2B LC101378.1
RVs/HoChiMinh.VNM/43.11-CRS AB745034.1
RVs/FukuokaC.JPN/27.11-2B AB824845.1
RVs/FukuokaC.JPN/34.11-2B AB824846.1
RVs/Fukuokacity.JPN/53.11 AB702684.1
74
RVs/HoChiMinh.VNM/26.11 AB706305.1
Rvs/Kobe.JPN/28.12/2-2B AB934137.1
RVs/Phetchaburi.THA/8.12/1-2B KT369580.1
Rvs/Kobe.JPN/47.12/2-2BCRS AB934147.1
Rvs/Kobe.JPN/42.12/1-2BCRS AB934145.1
Rvs/Kobe.JPN/31.12-2B AB934139.1
Rvs/Kobe.JPN/24.12-2B AB934135.1
Rvs/Kobe.JPN/16.12 AB934134.1
Rvs/Kobe.JPN/13.12-2B AB934131.1
Rvs/FukuokaC.JPN/24.12-2B AB824847.1
Rvi/Kobe.JPN/27.12-2B AB934142.1
Rvi/Kobe.JPN/21.12/2-2B AB934141.1
Rvs/Kobe.JPN/15.12-2B AB934130.1
Rvi/Kobe.JPN/22.13/2-2B AB934168.1
Rvs/Kobe.JPN/8.13-2B AB934152.1
2B-L1

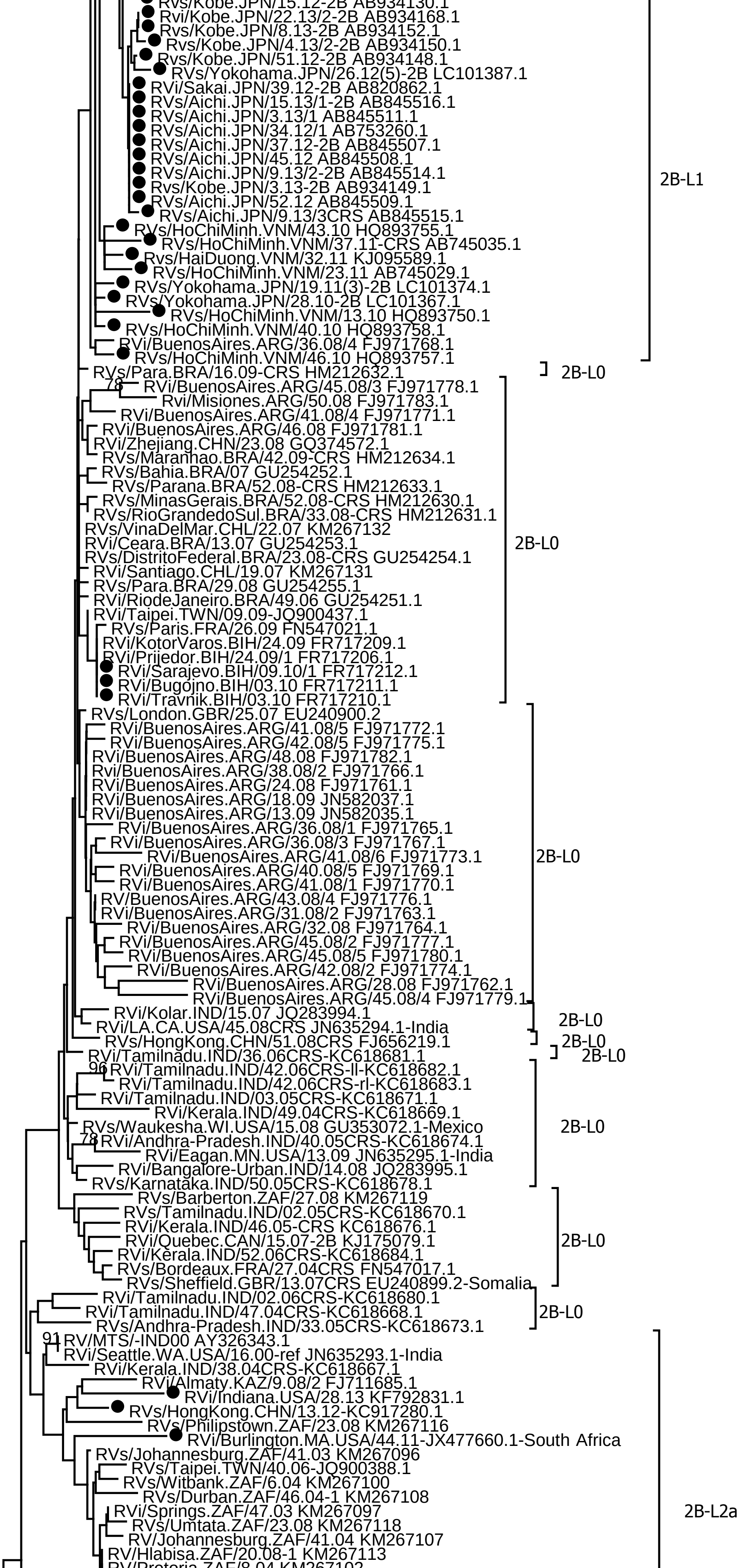

RVs/Kobe.JPN/15.12-2B AB934130.1
Rvi/Kobe.JPN/22.13/2-2B AB934168.1
Rvs/Kobe.JPN/8.13-2B AB934152.1
Rvs/Kobe.JPN/4.13/2-2B AB934150.1
Rvs/Kobe.JPN/51.12-2B AB934148.1
RVs/Yokohama.JPN/26.12(5)-2B LC101387.1
RVi/Sakai.JPN/39.12-2B AB820862.1
RVs/Aichi.JPN/15.13/1-2B AB845516.1
RVs/Aichi.JPN/3.13/1 AB845511.1
RVs/Aichi.JPN/34.12/1 AB753260.1
RVs/Aichi.JPN/37.12-2B AB845507.1
RVs/Aichi.JPN/45.12 AB845508.1
RVs/Aichi.JPN/9.13/2-2B AB845514.1
Rvs/Kobe.JPN/3.13-2B AB934149.1
RVs/Aichi.JPN/52.12 AB845509.1
RVs/Aichi.JPN/9.13/3CRS AB845515.1
2B-L1
RVs/HoChiMinh.VNM/43.10 HQ893755.1
RVs/HoChiMinh.VNM/37.11-CRS AB745035.1
Rvs/HaiDuong.VNM/32.11 KJ095589.1
RVs/HoChiMinh.VNM/23.11 AB745029.1
RVs/Yokohama.JPN/19.11(3)-2B LC101374.1
RVs/Yokohama.JPN/28.10-2B LC101367.1
RVs/HoChiMinh.VNM/13.10 HQ893750.1
RVs/HoChiMinh.VNM/40.10 HQ893758.1
RVi/BuenosAires.ARG/36.08/4 FJ971768.1
RVs/HoChiMinh.VNM/46.10 HQ893757.1
RVs/Para.BRA/16.09-CRS HM212632.1
2B-L0
78
RVi/BuenosAires.ARG/45.08/3 FJ971778.1
Rvi/Misiones.ARG/50.08 FJ971783.1
RVi/BuenosAires.ARG/41.08/4 FJ971771.1
RVi/BuenosAires.ARG/46.08 FJ971781.1
RVi/Zhejiang.CHN/23.08 GQ374572.1
RVs/Maranhao.BRA/42.09-CRS HM212634.1
RVs/Bahia.BRA/07 GU254252.1
RVs/Parana.BRA/52.08-CRS HM212633.1
RVs/MinasGerais.BRA/52.08-CRS HM212630.1
RVs/RioGrandedoSul.BRA/33.08-CRS HM212631.1
RVs/VinaDelMar.CHL/22.07 KM267132
RVi/Ceara.BRA/13.07 GU254253.1
RVs/DistritoFederal.BRA/23.08-CRS GU254254.1
RVi/Santiago.CHL/19.07 KM267131
RVs/Para.BRA/29.08 GU254255.1
RVi/RiodeJaneiro.BRA/49.06 GU254251.1
RVi/Taipei.TWN/09.09-JQ900437.1
RVs/Paris.FRA/26.09 FN547021.1
RVi/KotorVaros.BIH/24.09 FR717209.1
RVi/Prijedor.BIH/24.09/1 FR717206.1
RVi/Sarajevo.BIH/09.10/1 FR717212.1
RVi/Bugojno.BIH/03.10 FR717211.1
RVi/Travnik.BIH/03.10 FR717210.1
2B-L0
RVs/London.GBR/25.07 EU240900.2
RVi/BuenosAires.ARG/41.08/5 FJ971772.1
RVi/BuenosAires.ARG/42.08/5 FJ971775.1
RVi/BuenosAires.ARG/48.08 FJ971782.1
Rvi/BuenosAires.ARG/38.08/2 FJ971766.1
RVi/BuenosAires.ARG/24.08 FJ971761.1
RVi/BuenosAires.ARG/18.09 JN582037.1
RVi/BuenosAires.ARG/13.09 JN582035.1
RVi/BuenosAires.ARG/36.08/1 FJ971765.1
RVi/BuenosAires.ARG/36.08/3 FJ971767.1
RVi/BuenosAires.ARG/41.08/6 FJ971773.1
RVi/BuenosAires.ARG/40.08/5 FJ971769.1
RVi/BuenosAires.ARG/41.08/1 FJ971770.1
RV/BuenosAires.ARG/43.08/4 FJ971776.1
RVi/BuenosAires.ARG/31.08/2 FJ971763.1
RVi/BuenosAires.ARG/32.08 FJ971764.1
RVi/BuenosAires.ARG/45.08/2 FJ971777.1
RVi/BuenosAires.ARG/45.08/5 FJ971780.1
RVi/BuenosAires.ARG/42.08/2 FJ971774.1
RVi/BuenosAires.ARG/28.08 FJ971762.1
RVi/BuenosAires.ARG/45.08/4 FJ971779.1
2B-L0
RVi/Kolar.IND/15.07 JQ283994.1
RVi/LA.CA.USA/45.08CRS JN635294.1-India
2B-L0
RVs/HongKong.CHN/51.08CRS FJ656219.1
2B-L0
RVi/Tamilnadu.IND/36.06CRS-KC618681.1
2B-L0
96
RVi/Tamilnadu.IND/42.06CRS-ll-KC618682.1
RVi/Tamilnadu.IND/42.06CRS-rl-KC618683.1
RVi/Tamilnadu.IND/03.05CRS-KC618671.1
RVi/Kerala.IND/49.04CRS-KC618669.1
RVs/Waukesha.WI.USA/15.08 GU353072.1-Mexico
78
RVi/Andhra-Pradesh.IND/40.05CRS-KC618674.1
RVi/Eagan.MN.USA/13.09 JN635295.1-India
RVi/Bangalore-Urban.IND/14.08 JQ283995.1
RVs/Karnataka.IND/50.05CRS-KC618678.1
2B-L0
RVs/Barberton.ZAF/27.08 KM267119
RVs/Tamilnadu.IND/02.05CRS-KC618670.1
RVi/Kerala.IND/46.05-CRS KC618676.1
RVi/Quebec.CAN/15.07-2B KJ175079.1
RVi/Kerala.IND/52.06CRS-KC618684.1
RVs/Bordeaux.FRA/27.04CRS FN547017.1
RVs/Sheffield.GBR/13.07CRS EU240899.2-Somalia
2B-L0
RVi/Tamilnadu.IND/02.06CRS-KC618680.1
RVi/Tamilnadu.IND/47.04CRS-KC618668.1
RVs/Andhra-Pradesh.IND/33.05CRS-KC618673.1
2B-L0
91
RV/MTS/-IND00 AY326343.1
RVi/Seattle.WA.USA/16.00-ref JN635293.1-India
RVi/Kerala.IND/38.04CRS-KC618667.1
RVi/Almaty.KAZ/9.08/2 FJ711685.1
RVi/Indiana.USA/28.13 KF792831.1
RVs/HongKong.CHN/13.12-KC917280.1
RVs/Philipstown.ZAF/23.08 KM267116
RVi/Burlington.MA.USA/44.11-JX477660.1-South Africa
RVs/Johannesburg.ZAF/41.03 KM267096
RVs/Taipei.TWN/40.06-JQ900388.1
RVs/Witbank.ZAF/6.04 KM267100
RVs/Durban.ZAF/46.04-1 KM267108
RVi/Springs.ZAF/47.03 KM267097
RVs/Umtata.ZAF/23.08 KM267118
RV/Johannesburg.ZAF/41.04 KM267107
RV/Hlabisa.ZAF/20.08-1 KM267113
RV/Pretoria.ZAF/8.04 KM267102
2B-L2a

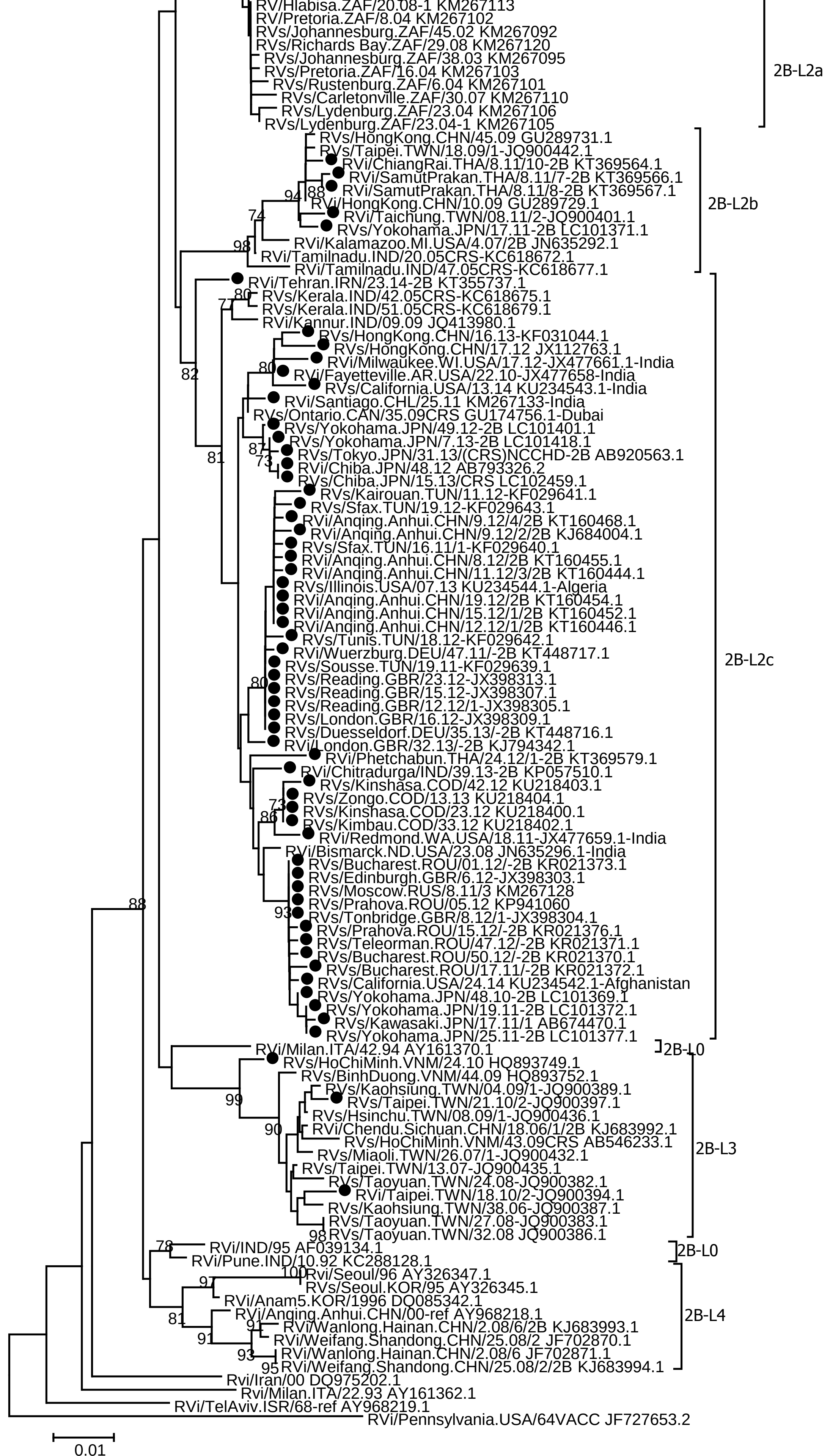

RV/Hlabisa.ZAF/20.08-1 KM267113
RV/Pretoria.ZAF/8.04 KM267102
RVs/Johannesburg.ZAF/45.02 KM267092
RVs/Richards Bay.ZAF/29.08 KM267120
RVs/Johannesburg.ZAF/38.03 KM267095
RVs/Pretoria.ZAF/16.04 KM267103
RVs/Rustenburg.ZAF/6.04 KM267101
RVs/Carletonville.ZAF/30.07 KM267110
RVs/Lydenburg.ZAF/23.04 KM267106
RVs/Lydenburg.ZAF/23.04-1 KM267105
2B-L2a
RVs/HongKong.CHN/45.09 GU289731.1
RVs/Taipei.TWN/18.09/1-JQ900442.1
RVi/ChiangRai.THA/8.11/10-2B KT369564.1
RVi/SamutPrakan.THA/8.11/7-2B KT369566.1
RVi/SamutPrakan.THA/8.11/8-2B KT369567.1
94
88
RVi/HongKong.CHN/10.09 GU289729.1
74
RVi/Taichung.TWN/08.11/2-JQ900401.1
RVs/Yokohama.JPN/17.11-2B LC101371.1
RVi/Kalamazoo.MI.USA/4.07/2B JN635292.1
98
RVi/Tamilnadu.IND/20.05CRS-KC618672.1
RVi/Tamilnadu.IND/47.05CRS-KC618677.1
2B-L2b
RVi/Tehran.IRN/23.14-2B KT355737.1
80
RVs/Kerala.IND/42.05CRS-KC618675.1
77
RVs/Kerala.IND/51.05CRS-KC618679.1
RVi/Kannur.IND/09.09 JQ413980.1
RVs/HongKong.CHN/16.13-KF031044.1
RVs/HongKong.CHN/17.12 JX112763.1
RVi/Milwaukee.WI.USA/17.12-JX477661.1-India
80
82
RVi/Fayetteville.AR.USA/22.10-JX477658-India
RVs/California.USA/13.14 KU234543.1-India
RVi/Santiago.CHL/25.11 KM267133-India
RVs/Ontario.CAN/35.09CRS GU174756.1-Dubai
RVs/Yokohama.JPN/49.12-2B LC101401.1
RVs/Yokohama.JPN/7.13-2B LC101418.1
87
81
RVs/Tokyo.JPN/31.13/(CRS)NCCHD-2B AB920563.1
73
RVi/Chiba.JPN/48.12 AB793326.2
RVs/Chiba.JPN/15.13/CRS LC102459.1
RVs/Kairouan.TUN/11.12-KF029641.1
RVs/Sfax.TUN/19.12-KF029643.1
RVi/Anqing.Anhui.CHN/9.12/4/2B KT160468.1
RVi/Anqing.Anhui.CHN/9.12/2/2B KJ684004.1
RVs/Sfax.TUN/16.11/1-KF029640.1
RVi/Anqing.Anhui.CHN/8.12/2B KT160455.1
RVi/Anqing.Anhui.CHN/11.12/3/2B KT160444.1
RVs/Illinois.USA/07.13 KU234544.1-Algeria
RVi/Anqing.Anhui.CHN/19.12/2B KT160454.1
RVi/Anqing.Anhui.CHN/15.12/1/2B KT160452.1
RVi/Anqing.Anhui.CHN/12.12/1/2B KT160446.1
RVs/Tunis.TUN/18.12-KF029642.1
RVi/Wuerzburg.DEU/47.11/-2B KT448717.1
RVs/Sousse.TUN/19.11-KF029639.1
80
RVs/Reading.GBR/23.12-JX398313.1
RVs/Reading.GBR/15.12-JX398307.1
RVs/Reading.GBR/12.12/1-JX398305.1
RVs/London.GBR/16.12-JX398309.1
RVs/Duesseldorf.DEU/35.13/-2B KT448716.1
RVi/London.GBR/32.13/-2B KJ794342.1
RVi/Phetchabun.THA/24.12/1-2B KT369579.1
RVi/Chitradurga/IND/39.13-2B KP057510.1
RVs/Kinshasa.COD/42.12 KU218403.1
RVs/Zongo.COD/13.13 KU218404.1
73
RVs/Kinshasa.COD/23.12 KU218400.1
86
RVs/Kimbau.COD/33.12 KU218402.1
RVi/Redmond.WA.USA/18.11-JX477659.1-India
RVi/Bismarck.ND.USA/23.08 JN635296.1-India
RVs/Bucharest.ROU/01.12/-2B KR021373.1
RVs/Edinburgh.GBR/6.12-JX398303.1
RVs/Moscow.RUS/8.11/3 KM267128
RVs/Prahova.ROU/05.12 KP941060
93
RVs/Tonbridge.GBR/8.12/1-JX398304.1
RVs/Prahova.ROU/15.12/-2B KR021376.1
RVs/Teleorman.ROU/47.12/-2B KR021371.1
RVs/Bucharest.ROU/50.12/-2B KR021370.1
RVs/Bucharest.ROU/17.11/-2B KR021372.1
RVs/California.USA/24.14 KU234542.1-Afghanistan
RVs/Yokohama.JPN/48.10-2B LC101369.1
RVs/Yokohama.JPN/19.11-2B LC101372.1
RVs/Kawasaki.JPN/17.11/1 AB674470.1
RVs/Yokohama.JPN/25.11-2B LC101377.1
2B-L2c
88
RVi/Milan.ITA/42.94 AY161370.1
2B-L0
RVs/HoChiMinh.VNM/24.10 HQ893749.1
RVs/BinhDuong.VNM/44.09 HQ893752.1
RVs/Kaohsiung.TWN/04.09/1-JQ900389.1
RVs/Taipei.TWN/21.10/2-JQ900397.1
99
RVs/Hsinchu.TWN/08.09/1-JQ900436.1
90
RVi/Chendu.Sichuan.CHN/18.06/1/2B KJ683992.1
RVs/HoChiMinh.VNM/43.09CRS AB546233.1
RVs/Miaoli.TWN/26.07/1-JQ900432.1
RVs/Taipei.TWN/13.07-JQ900435.1
RVs/Taoyuan.TWN/24.08-JQ900382.1
RVi/Taipei.TWN/18.10/2-JQ900394.1
RVs/Kaohsiung.TWN/38.06-JQ900387.1
RVs/Taoyuan.TWN/27.08-JQ900383.1
98
RVs/Taoyuan.TWN/32.08 JQ900386.1
2B-L3
78
RVi/IND/95 AF039134.1
RVi/Pune.IND/10.92 KC288128.1
2B-L0
100
RVi/Seoul/96 AY326347.1
97
RVs/Seoul.KOR/95 AY326345.1
RVi/Anam5.KOR/1996 DQ085342.1
81
RVi/Anqing.Anhui.CHN/00-ref AY968218.1
91
RVi/Wanlong.Hainan.CHN/2.08/6/2B KJ683993.1
91
RVi/Weifang.Shandong.CHN/25.08/2 JF702870.1
93
RVi/Wanlong.Hainan.CHN/2.08/6 JF702871.1
95
RVi/Weifang.Shandong.CHN/25.08/2/2B KJ683994.1
2B-L4
Rvi/Iran/00 DQ975202.1
Rvi/Milan.ITA/22.93 AY161362.1
RVi/TelAviv.ISR/68-ref AY968219.1
RVi/Pennsylvania.USA/64VACC JF727653.2
0.01
